# A count-based model for delineating cell–cell interactions in spatial transcriptomics data

**DOI:** 10.1093/bioinformatics/btae219

**Published:** 2024-06-28

**Authors:** Hirak Sarkar, Uthsav Chitra, Julian Gold, Benjamin J Raphael

**Affiliations:** Department of Computer Science, Princeton University, Princeton, NJ, 08540, United States; Ludwig Cancer Institute, Princeton Branch, Princeton University, Princeton, NJ, 08540, United States; Department of Computer Science, Princeton University, Princeton, NJ, 08540, United States; Department of Computer Science, Princeton University, Princeton, NJ, 08540, United States; Center for Statistics and Machine Learning, Princeton University, Princeton, NJ, 08540, United States; Department of Computer Science, Princeton University, Princeton, NJ, 08540, United States

## Abstract

**Motivation:**

Cell–cell interactions (CCIs) consist of cells exchanging signals with themselves and neighboring cells by expressing ligand and receptor molecules and play a key role in cellular development, tissue homeostasis, and other critical biological functions. Since direct measurement of CCIs is challenging, multiple methods have been developed to infer CCIs by quantifying correlations between the gene expression of the ligands and receptors that mediate CCIs, originally from bulk RNA-sequencing data and more recently from single-cell or spatially resolved transcriptomics (SRT) data. SRT has a particular advantage over single-cell approaches, since ligand–receptor correlations can be computed between cells or spots that are physically close in the tissue. However, the transcript counts of individual ligands and receptors in SRT data are generally low, complicating the inference of CCIs from expression correlations.

**Results:**

We introduce Copulacci, a count-based model for inferring CCIs from SRT data. Copulacci uses a Gaussian copula to model dependencies between the expression of ligands and receptors from nearby spatial locations even when the transcript counts are low. On simulated data, Copulacci outperforms existing CCI inference methods based on the standard Spearman and Pearson correlation coefficients. Using several real SRT datasets, we show that Copulacci discovers biologically meaningful ligand–receptor interactions that are lowly expressed and undiscoverable by existing CCI inference methods.

**Availability and implementation:**

Copulacci is implemented in Python and available at https://github.com/raphael-group/copulacci.

## 1 Introduction

Cell–cell interactions (CCIs) are fundamental to many biological processes in multicellular organisms including cellular differentiation (Amprino 1953, Kirouac *et al.* 2010), homeostasis (Wu *et al.* 2018), immune response (Baccin *et al.* 2020), wound healing (Jin *et al.* 2021), and response to disease condition (Kveler *et al.* 2018). For example, interactions between tumor cells and nearby endothelial cells lead to the formation of blood vessels in the tumor microenvironment (Rivera and Bergers 2015), enabling tumor growth and metastasis (Nishida-Aoki and Gujral 2019, Lu *et al.* 2012). Cells communicate with each other by exchanging biochemical signals (Skinner 1991) where a ligand emitted by one cell binds to a receptor on the surface of another cell. Thus, identifying the ligand and receptor molecules that mediate CCIs inside a tissue is an important problem with many biological applications (Miller and Lappin 2020).

Direct measurement of CCIs—e.g. through biophysical imaging, immunofluorescence staining (Leineweber 2023), and other technologies—is technologically challenging and does not readily scale to thousands of ligand and receptor molecules inside a single tissue. Thus, an alternative approach is to infer CCIs from the co-expression of ligand and receptor in bulk RNA-seq (Choi *et al.* 2015) or single-cell RNA-sequencing (scRNA-seq) (Hou *et al.* 2020, Cabello-Aguilar *et al.* 2020, Hu *et al.* 2021, Wang *et al.* 2019). These methods typically rely on a set of ligands and receptors from the manually curated database (Türei *et al.* 2021, Efremova *et al.* 2020, Jin *et al.* 2021) of validated or predicted CCIs.

An important limitation of using scRNA-seq data to infer CCIs is that scRNA-seq technologies dissociate cells before measurement, thus losing the spatial positions of individual cells, which are a key constraint on communicating cells. As a result, scRNA-seq-based methods for CCI inference tend to infer many false positive interactions, as they are unable to distinguish spatially adjacent cells that are interacting versus cells that are far from one another and have correlated ligand and receptor expressions (Bafna *et al.* 2023). Recent developments in spatially resolved transcriptomics (SRT) technologies (Williams *et al.* 2022, Rao *et al.* 2021) have led to the collection of high-throughput transcriptomics measurements together with the spatial locations of individual cells, enabling potentially more accurate inference of CCIs.

Several computational methods have been developed to infer CCIs from SRT data, generally following one of two different approaches. The first approach is to identify ligand–receptor pairs with a large value of a spatially aware co-expression score (Miller *et al.* 2021, Li *et al.* 2023, Pham *et al.* 2023, Dries *et al.* 2021) which is typically based on the Pearson correlation coefficient or the Moran’s I autocorrelation measure used in geospatial statistics (Getis and Ord 1992). However, such co-expression scores are challenged by the sparsity of current SRT technologies, and thus these methods struggle to identify interacting ligands and receptors with low unique molecular identifier (UMI) counts. Moreover, these methods identify only putative interacting ligands and receptors and do not identify the specific regions of a tissue where the interactions take place. The second approach, which has been followed by a few recent methods, is to explicitly model the biological cell signaling process inside a tissue, e.g. using Gaussian processes (Arnol *et al.* 2019) or optimal transport (Cang and Nie 2020, Cang *et al.* 2023). However, these methods must make broad assumptions about the biophysical process of inter-cellular communication, and as a result, are computationally inefficient. Moreover, none of the existing CCI inference methods account for the fact that SRT measures discrete UMI counts, as all of these approaches transform the counts to continuous values. However, many papers in the single-cell analysis literature have shown that such transformations introduce unwanted zero inflation and other biases in downstream analyses (Townes *et al.* 2019, Booeshaghi and Pachter 2021).

We introduce Copulacci, a copula-based method for inferring CCIs from sparse SRT data. We model the bivariate distribution of expression counts of each ligand and receptor using copulas, which encode interactions between random variables while allowing for arbitrary marginals. In particular, we use a bivariate Gaussian copula distribution, a widely used and interpretable distribution, and we use Poisson marginals to account for the discrete and sparse UMI counts in SRT data. We derive a computationally efficient optimization algorithm to maximize the likelihood of our model. The estimated parameters of our model reveal CCIs, i.e. which ligands and receptors are interacting. Moreover, Copulacci computes an interaction score for a ligand–receptor pair between pairs of nearby spatial locations, revealing the spatial regions where CCIs occur.

On simulated data with varying amounts of data sparsity, Copulacci yields demonstrably more accurate identification of CCIs compared to existing CCI inference methods, including the Pearson and Spearman correlation coefficients, with Copulacci having better performance on sparser data. The improved performance of Copulacci on sparse data is because Copulacci models the *distribution* of ligand and receptor co-expression, in contrast to conventional CCI inference methods which only learn a *point estimate* for the dependency between ligand and receptor co-expression. On SRT data from a human breast cancer (10x Visium), mouse cortex (seqFISH+), and mouse embryo (Stereo-seq), we show that Copulacci discovers many biologically relevant CCIs that are not identified by existing approaches due to low expression of ligands and/or receptors.

## 2 Materials and methods

### 2.1 Cell–cell interaction inference problem

We start by formalizing the problem of identifying CCIs from SRT data. Suppose we are given UMI counts $\ell =[\ell_{i}]\in R_{\geq 0}^{N}$ and $r=[r_{i}]\in R_{\geq 0}^{N}$ for a ligand and receptor, respectively, across *N* spatial locations (spots) in a tissue slice, as well as the spatial location $s_{i}\in R^{2}$ of each spot i=1,…,N. We emphasize that each spot *i* has two measurements: a ligand expression $\ell_{i}$*and* a receptor expression $r_{i}$.

We describe the potential CCIs with a directed graph G=(V,E). The vertices V={1,…,N} represent the spots and each directed edge (i,j)∈E represents a potential CCI where a ligand expressed in spot *i* may bind with a receptor expressed in spot *j*. Typically the directed graph *G* is *symmetric*, meaning that (i,j)∈E implies (j,i)∈E; that is, if a ligand from spot *i* is able to bind with a receptor in spot *j*, then a ligand in spot *j* is also able to bind with a receptor in spot *i*. For this reason, one may equivalently describe the potential CCIs with an *undirected* graph, where an undirected edge (i,j) in an undirected graph corresponds to two directed edges (i,j),(j,i)∈E in a directed graph *G*. We will use the undirected graph representation when it is clear from the context.

The inclusion of an edge (i,j)∈E typically depends on the interaction range of the ligand and receptor and the physical distance between spots *i* and *j*. For example, most signaling occurs between cells that are within 100–200 μm of one another, and thus the edges *E* connect spots that are at most 200 μm apart. In SRT data measured using the 10x Genomics Visium technology, each spot lies on a hexagonal grid and adjacent spots are 100 μm apart, and so one natural choice for the graph *G* is a connected subgraph of the hexagonal lattice. The graph may also be weighted, where each edge (i,j)∈E has a weight $w_{i,j}$ reflecting the physical distance between spots *i* and *j*.

The standard approach to identify CCIs mediated by a ligand and receptor is to test whether the ligand expression $\ell_{i}$ and receptor expression $r_{j}$ across spatially adjacent spots (i,j)∈E are dependent on each other or not. More formally, given samples ${\left\{(\ell_{i},r_{j})\right\}}_{(i,j)\in E}$ of a pair of random variables (L,R) representing a ligand *L* and a receptor *R*, we aim to estimate the “dependency” between *L* and *R*, where we use quotes to indicate different possible choices for measuring the dependency.

We formalize this task as the following computational problem.

#### 2.1.1 CCI inference problem

*Given a spatial adjacency graph* G=(V,E)*, and samples* ${\left\{(\ell_{i},r_{j})\right\}}_{(i,j)\in E}$*of a bivariate random variable* (L,R)*, estimate a dependency score between L and R.*

Nearly all existing CCI inference methods solve the CCI inference problem (CCIIP) with the dependency score corresponding to the following correlation coefficient:

(1) $$\rho_{\text{spatial}}=c\cdot \frac{\sum_{(i,j)\in E}w_{i,j}(\ell_{i}\;-\;\bar{\ell })(r_{j}\;-\;\bar{r})}{\sqrt{\sum_{i}{\left(\ell_{i}\;-\;\bar{\ell }\right)}^{2}}\sqrt{\sum_{j}{\left(r_{j}\;-\;\bar{r}\right)}^{2}}},$$

for some choice of edge weights $w_{i,j}$ and normalizing constant *c*, where $\bar{\ell },\bar{r}$ are the mean expression of ligand and receptor, respectively.

We call $\rho_{\text{spatial}}$ a *generalized Pearson correlation measure*, as $\rho_{\text{spatial}}$ is equal to the Pearson correlation coefficient when c=1 and *G* is an unweighted graph, i.e. $w_{i,j}=1$ for all edges (i,j)∈E. The generalized Pearson correlation measure $\rho_{\text{spatial}}$ is sometimes called a bivariate Moran’s I measure in the single-cell literature, as it bears close similarity to the Moran’s I statistic used to measure autocorrelation in geospatial statistics. For example, MERINGUE (Miller *et al.* 2021) solves the CCIIP with a generalized Pearson correlation measure $\rho_{\text{SCC}}$, which they call a spatial cross-correlation measure, where *G* is an unweighted graph with an edge between two spots if they are adjacent in a Delauney triangulation and $c=\frac{N}{\left|E\right|}$. SpatialDM (Li *et al.* 2023) solves the CCIIP using the generalized Pearson correlation measure $\rho_{\text{SpatialDM}}$ where c=1 and the spatial adjacency graph *G* is a complete graph with edge weights $w_{i,j}\propto \;\text{exp}\;\left(-\frac{d_{i,j}}{2\sigma^{2}}\right)$, where $d_{i,j}=||s_{i}-s_{j}|{|}_{2}$ is the physical distance between spot *i* and spot *j*.

A major limitation of the generalized Pearson correlation measure $\rho_{\text{spatial}}$ is that it does not account for the low coverage and sparsity of current SRT technologies. For example, the standard Pearson correlation coefficient is known to have reduced power for non-normally distributed data (Bishara and Hittner 2012), and the distribution of sparse UMI counts is not close to being normally distributed. More generally, the underlying issue is that nearly all existing approaches for identifying CCIs derive a dependency score for each ligand–receptor pair that does not model the joint *distribution* of ligand and receptor expression values.

A natural approach for identifying CCIs would be to derive a *bivariate* count-based distribution to model the co-expression of both a ligand and receptor gene. To this end, several count-based distributions, including Poisson and negative binomial distributions, have been used to model sparse UMI counts of *individual* genes in single-cell RNA-sequencing data (Townes *et al.* 2019, O’Hara and Kotze 2010, Hafemeister and Satija 2019). However, there are numerous challenges in extending such a univariate count distribution to a bivariate distribution. In particular, unlike the multivariate normal distribution which has a well-established canonical form, the multivariate Poisson and negative binomial distributions lack a universally accepted definition (Inouye *et al.* 2017). For example, Campbell (1934) defined a bivariate Poisson distribution as a summation of independent Poisson variables. However, this definition only permits positive dependency between variables. Other definitions of bivariate count distributions capable of accommodating both positive and negative dependency, such as the bivariate negative binomial distributions defined by Subrahmaniam and Subrahmaniam (1973) and Mitchell and Paulson (1981), require computationally intensive inference procedures for parameter estimation (Inouye *et al.* 2017).

**Figure 1.**
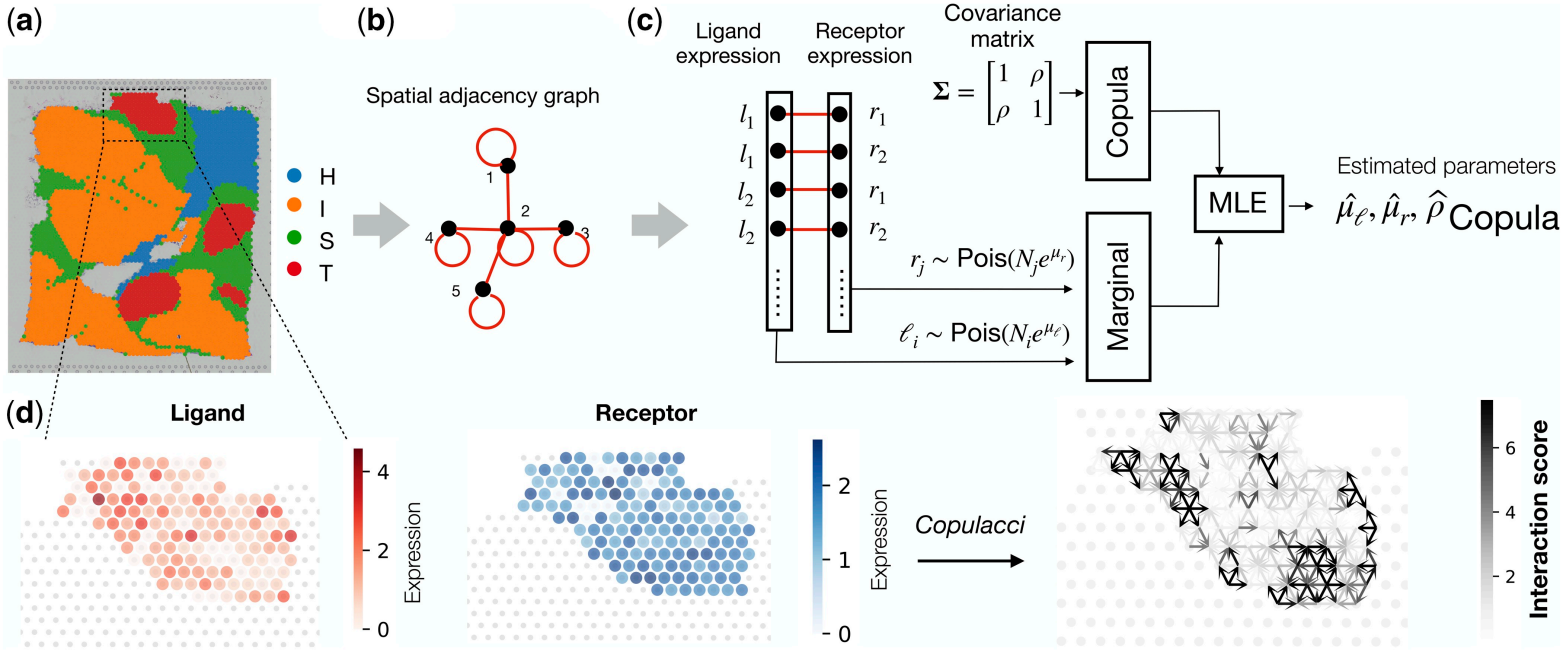
Overview of Copulacci. **(a)** A breast cancer SRT dataset with four cell type labels: tumor (T), surrounding tumor (S), invasive (I), and healthy (H). **(b)** Spatial adjacency graph G=(V,E) where edges *E* connect *physically adjacent* spots, i.e. spots within a predefined distance from one another. **(c)** Copulacci uses a bivariate Gaussian copula with Poisson marginals to model the distribution of observed ligand and receptor expression values ${\left\{(\ell_{i},r_{j})\right\}}_{(i,j)\in E}$ across each edge (i,j)∈E in the spatial adjacency graph *G*. The copula distribution is parameterized by the correlation coefficient ρ of the covariance matrix, which we call the copula correlation coefficient, and the mean expression $\mu_{\ell },\mu_{r}$ of the ligand and receptor, respectively. Copulacci computes the maximum likelihood estimators (MLEs) $\hat{\rho }$, ${\hat{\mu }}_{\ell }$, ${\hat{\mu }}_{r}$ of the copula correlation coefficient, mean ligand expression, and mean receptor expression, respectively. **(d)** (Left) Ligand and receptor expression values for spots with tumor (T) cell type label. (Right) Copulacci estimates model parameters $\hat{\rho }$, ${\hat{\mu }}_{\ell }$, ${\hat{\mu }}_{r}$ and computes an *interaction score* (quantified by Mahalanobis distance from the estimated copula distribution) which indicates the strength and direction of cell–cell interactions between adjacent spots.

To address these challenges we introduce Copulacci (Fig. 1), a framework for using *copula* distributions to model the joint distribution of ligand expression and receptor expression. Copulas are a general framework for modeling multivariate distributions as they can model arbitrary count distributions including Poisson or negative binomial distributions; include both positive and negative correlations; and are efficient to fit in practice (see Inouye *et al.* 2017 for details). We note that copula distributions have previously been used by the scDesign framework to model and simulate UMI count matrices in single-cell transcriptomics data (Sun *et al.* 2021, Song *et al.* 2023).

### 2.2 A bivariate copula model for cell–cell interactions

Copulacci models the joint distribution $P(\ell_{i},r_{j})$ of adjacent ligand and receptor expressions using a *bivariate copula*. A bivariate copula is a function that encodes dependency relationships (positive or negative) between two random variables *L* and *R*, while allowing for arbitrary marginal distributions P(L),P(R) on the random variables.

Formally, a bivariate copula $C:{\left[0,1\right]}^{2}\to [0,1]$ is a function that is equal to the joint cumulative distribution function (CDF) of two random variables with uniform marginals. The benefit of copulas comes from Sklar’s theorem, which states that *any* bivariate distribution on random variables (L,R) with joint CDF F(ℓ,r) satisfies

(2) $$F(\ell ,r)=C(F_{L}(\ell ),F_{R}(r)),$$

for a copula function C(ℓ,r), where $F_{L}(\ell )$, $F_{R}(r)$ are the marginal CDFs of the respective random variables *L*, *R*.

From Equation (2), in order to define the joint distribution $P(\ell_{i},r_{j})$ of ligand and receptor UMI counts in adjacent spots (i,j)∈E, which is equivalent to defining the joint CDF F(ℓ,r), we must specify (1) a copula function C(ℓ,r) and (2) the marginal distribution CDFs $F_{L}(\ell_{i}),F_{R}(r_{j})$ of the ligand and receptor expression values, respectively.

#### 2.2.1 Gaussian copula

We use a bivariate Gaussian copula function $C_{\Sigma }(\ell ,r)=\Phi_{N(0,\Sigma )}(\Phi_{N(0,1)}^{-1}(F_{L}(\ell )),\Phi_{N(0,1)}^{-1}(F_{R}(r)))$, where $\Phi_{N(0,1)}$ is the CDF of a standard normal distribution and $\Phi_{N(0,\Sigma )}$ is the CDF of a bivariate Gaussian with mean zero and covariance matrix $\Sigma \in R^{2\times 2}$. Gaussian copulas are often used in practice due to their flexibility and interpretability (Inouye *et al.* 2017, Madsen, 2009).

For our applications, we use a Gaussian copula $C_{\Sigma_{\rho }}$ with covariance matrix $\Sigma_{\rho }=\left(\begin{array}{cc} 1 & \rho \\ \rho & 1 \end{array}\right)$. The Gaussian copula $C_{\Sigma_{\rho }}$ is parametrized by a parameter ρ which describes the correlation between the ligand and receptor expression values. For this reason, we call ρ the *copula correlation coefficient*. For notational simplicity, we write $C_{\Sigma_{\rho }}$ as $C_{\rho }$.

#### 2.2.2 Marginal distributions

We model the marginal distributions of the ligand and receptor expression values $\ell_{i},r_{j}$, respectively, with Poisson distributions of the form $\ell_{i}∼\text{Pois}(N_{i}e^{\mu_{\ell }})$ and $r_{j}∼\text{Pois}(N_{j}e^{\mu_{r}})$, where $N_{i}$ is the total UMI count at spot *i* and $\mu_{\ell },\mu_{r}$ are the (normalized) mean expression values for genes ℓ,r, respectively. Poisson distributions have been previously used to model sparse UMI counts in single-cell and SRT (Townes *et al.* 2019, Ma *et al.* 2022, Chitra *et al.* 2023). However, we emphasize that one may use other count-based distributions too, e.g. negative binomial or multinomial distributions (Townes *et al.* 2019, Baker 1994). We use $F_{L}(\ell_{i}|\mu_{\ell })$ and $F_{R}(r_{i}|\mu_{r})$ to refer to the marginal CDFs of the Poisson distributions for the ligand and receptor expressions, respectively.

#### 2.2.3 Maximum likelihood

From Equation (2), the joint CDF over the observed ligand, receptor expressions $\ell_{i},r_{j}$ for each pair (i,j)∈E of adjacent spots are given by

(3) $$F(\ell_{i},r_{j})=C_{\rho }(F_{L}(\ell_{i}\;|\;\mu_{\ell }),F_{R}(r_{j}\;|\;\mu_{r})).$$

Subsequently, the corresponding joint probability distribution $P(\ell_{i},r_{j})$ is obtained by differentiating $C_{\rho }$,

(4) $$P(\ell_{i},r_{j})=c_{\rho }(F_{L}(\ell_{i}|\mu_{\ell }),F_{R}(r_{j}|\mu_{r}))P(\ell_{i}|\mu_{\ell })P(r_{j}|\mu_{r})$$

We estimate the parameters $\rho ,\mu_{\ell },\mu_{r}$ by maximizing the log-likelihood of the data across all adjacent spots (i,j)∈E:

(5) $${\hat{\rho }}_{\text{Copula}},{\hat{\mu }}_{\ell },{\hat{\mu }}_{r}=\underset{\rho ,\mu_{\ell },\mu_{r}}{\text{argmax}}\;\text{log}\;P({\left\{(\ell_{i},r_{j})\right\}}_{(i,j)\in E}|\rho ,\mu_{\ell },\mu_{r})=\underset{\rho ,\mu_{\ell },\mu_{r}}{\text{argmax}}\sum_{(i,j)\in E}(\text{log}\;P(\ell_{i}|\mu_{\ell })+\text{log}\;P(r_{j}|\mu_{r})+c_{\rho }(F_{L}(\ell_{i}|\mu_{\ell }),F_{R}(r_{j}|\mu_{r})))=\underset{\rho ,\mu_{\ell },\mu_{r}}{\text{argmax}}\;\text{log}\;L(\ell ,r,G|\rho ,\mu_{\ell },\mu_{r})$$

We use the estimated parameter ${\hat{\rho }}_{\text{Copula}}$, i.e. the estimated copula correlation coefficient, as our dependency score for solving the CCIIP.

#### 2.2.4 Identifiability

Sklar’s theorem states that any *continuous* multivariate distribution has a unique decomposition as a product of a copula function and its marginals. However, for discrete distributions, this decomposition may not be unique, i.e. there exist multiple different ways to write a discrete distribution as a product of a copula function and its marginals (Inouye *et al.* 2017). This non-identifiability may lead to inaccurate estimates of the copula correlation coefficient ${\hat{\rho }}_{\text{Copula}}$ (Genest and Nešlehová, 2007).

One standard approach to account for this unidentifiability is with a *distributional transform* (Rüschendorf and Rüschendorf 2013), which involves transforming a discrete marginal distribution $P(\ell_{i})$ into a continuous distribution by “smoothing” out the CDF of the distribution. We specifically follow the distributional transform approach described by Kazianka and Pilz (2010); see Supplementary Appendix and Inouye *et al.* (2017) , Kazianka and Pilz (2010) for details.

For ligand–receptor pairs where both the ligand and the receptor have low (or zero) expression across many spots, the maximum likelihood estimator (MLE) (5) may yield multiple values of the correlation ρ; e.g. if the log-likelihood $\text{log}\;L(\rho ,\mu_{\ell },\mu_{r}|\ell ,r,G)$ is a *non-convex* function of the correlation ρ. Thus, we exclude from our analysis ligand–receptor pairs where the log-likelihood function $\text{log}\;L(\rho ,\mu_{\ell },\mu_{r}|\ell ,r,G)$ is not a convex function of ρ, i.e. the log-likelihood has a non-positive second derivative as a function of ρ. Specifically, we exclude ligand–receptor pairs where $\frac{d^{2}f}{d\rho^{2}}{|}_{\rho =\rho_{0}}\;\leq \;0$ for some $-1\;\leq \;\rho_{0}\;\leq \;1$, where $f(\rho )=\text{log}\;L(\rho ,{\hat{\mu }}_{\ell },{\hat{\mu }}_{r}|\ell ,r,G)$ is the log-likelihood function where the ligand and receptor mean expression values $\mu_{\ell },\mu_{r}$, respectively, are set equal to their MLEs from (5) with fixed ρ=0.

### 2.3 Cell type-specific CCIs

When each spot in the SRT data is annotated with a cell type label, we formulate a *cell type-specific* CCIIP for identifying CCIs between two specific cell types. Specifically, given a cell type label $\gamma_{i}$ for each spot i=1,…,N, we identify CCIs between cell types *c* and c′ by solving the CCIIP on the graph $G_{c,c\prime }=(V_{c,c\prime },E_{c,c\prime })$ where the vertices $V_{c,c\prime }=\{i:\gamma_{i}=c\;\text{or}\;\gamma_{i}=c\prime \}$ are the spots with cell type label *c* or c′, and the edges $E_{c,c\prime }=\{(i,j)\in E:\gamma_{i}=c,\gamma_{j}=c\prime \;\text{or}\;\gamma_{i}=c\prime ,\gamma_{j}=c\}$ connect a cell with label *c* to a cell with label c′. We note that for technologies with low spatial resolution, such as 10x Visium, a spot is often annotated with a single cell type even though technically a spot may contain multiple cell types.

### 2.4 Statistical significance

After estimating the copula correlation coefficient ${\hat{\rho }}_{\text{Copula}}$, we aim to test whether the estimated coefficient ${\hat{\rho }}_{\text{Copula}}$ is larger (in absolute value) than expected by chance when cells are randomly arranged in the tissue. We formalize this with a *permutation test*, where we define the null distribution of correlation values ρ as the distribution of correlation coefficient measures ${\hat{\rho }}_{\text{Copula},G^{\prime }}$ estimated using graph $G^{\prime }$, where $G^{\prime }$ is obtained by permuting the spots in the spatial adjacency graph *G*; that is, $G^{\prime }=(V,E^{\prime })$ where (i,j)∈E if and only if $(\pi (i),\pi (j))\in E^{\prime }$ for a fixed permutation π on the vertices *V*.

We uniformly sample graphs $G^{\prime }$ by permuting the vertices (spots) in the spatial adjacency graph *G*, and we estimate the *p*-value as the fraction of graphs $G^{\prime }$ where $\left|{\hat{\rho }}_{\text{Copula},G^{\prime }}|>|\rho \right|$. We control the false discovery rate using the Benjamini-Hochberg procedure (Benjamini and Hochberg 1995). We emphasize that for the cell type-specific CCIIP, which is the problem that we solve in many of our applications, our permutation test only permutes spots of a given cell type(s).

### 2.5 CCI visualization

We use the estimated distribution $P(\ell_{i},r_{j})$ to identify spatial regions where ligand–receptor interactions are taking place. We define the *interaction score* between two adjacent spatial locations with ligand and receptor expression values $\ell_{i},r_{j}$ as the Mahalanobis distance $z_{ij}^{T}{\hat{\Sigma }}_{{\hat{\rho }}_{\text{Copula}}}^{-1}z_{ij}$ between the vector $z_{ij}=[\Phi_{N(0,1)}^{-1}(F_{L}(\ell_{i})),\Phi_{N(0,1)}^{-1}(F_{R}(r_{j}))]$ and the bivariate Gaussian distribution $N(0,{\hat{\Sigma }}_{{\hat{\rho }}_{\text{Copula}}})$. Here, $z_{ij}$ is a transformation of the ligand–receptor expression values $\left[\ell_{i},r_{j}\right]$ to the same scale as the estimated bivariate Gaussian distribution $N(0,{\hat{\Sigma }}_{{\hat{\rho }}_{\text{Copula}}})$. Intuitively, a larger Mahalanobis distance for spatial locations *i*, *j* implies that the ligand and receptor expression values $\ell_{i},r_{j}$ are substantially larger or smaller than the mean, indicating a potential interaction.

### 2.6 Data pre-processing and implementation

For SRT data measured with 10x Genomics Visium (10x Genomics) (resp. Stereo-seq; Chen *et al.* 2022), where the spots are organized on a hexagonal (resp. rectangular) grid, then the spatial adjacency graph *G* is a hexagonal (resp. rectangular) grid graph. For SRT data where the spots are organized in an irregular fashion, e.g. SRT measured using seqFISH+ (Eng *et al.* 2019), we use Squidpy (Palla *et al.* 2022) to construct a spatial neighborhood graph using a user-defined distance threshold. In all cases, we also include “self-loops” in the graph G=(V,E), i.e. edges (i,i)∈E from a spot *i* to itself. These self-loops account for SRT data where a spot may consist of multiple cells, e.g. data measured with 10x Genomics Visium, and additionally, they describe autocrine signaling, where a ligand of a cell binds to a receptor of the same cell.

Following Li *et al.* (2023) and Cang *et al.* (2023), Copulacci uses a set of candidate ligand–receptor pairs specified in CellChatDB (Jin *et al.* 2021) and further filters out the ligands and receptors that are not expressed in a fixed number of spots using the same filtering criteria as Li *et al.* (2023). Furthermore, the CellChatDB database often contains *heteromeric* ligands and/or receptors, meaning the ligand/receptor is a complex consisting of multiple genes. In such cases, we obtain a single expression value by summing the expression values of each gene in the complex. Since Copulacci directly models count data, we do not perform any numerical transformation of the data such as log-normalization.

## 3 Results

### 3.1 Simulated count data

**Figure 2.**
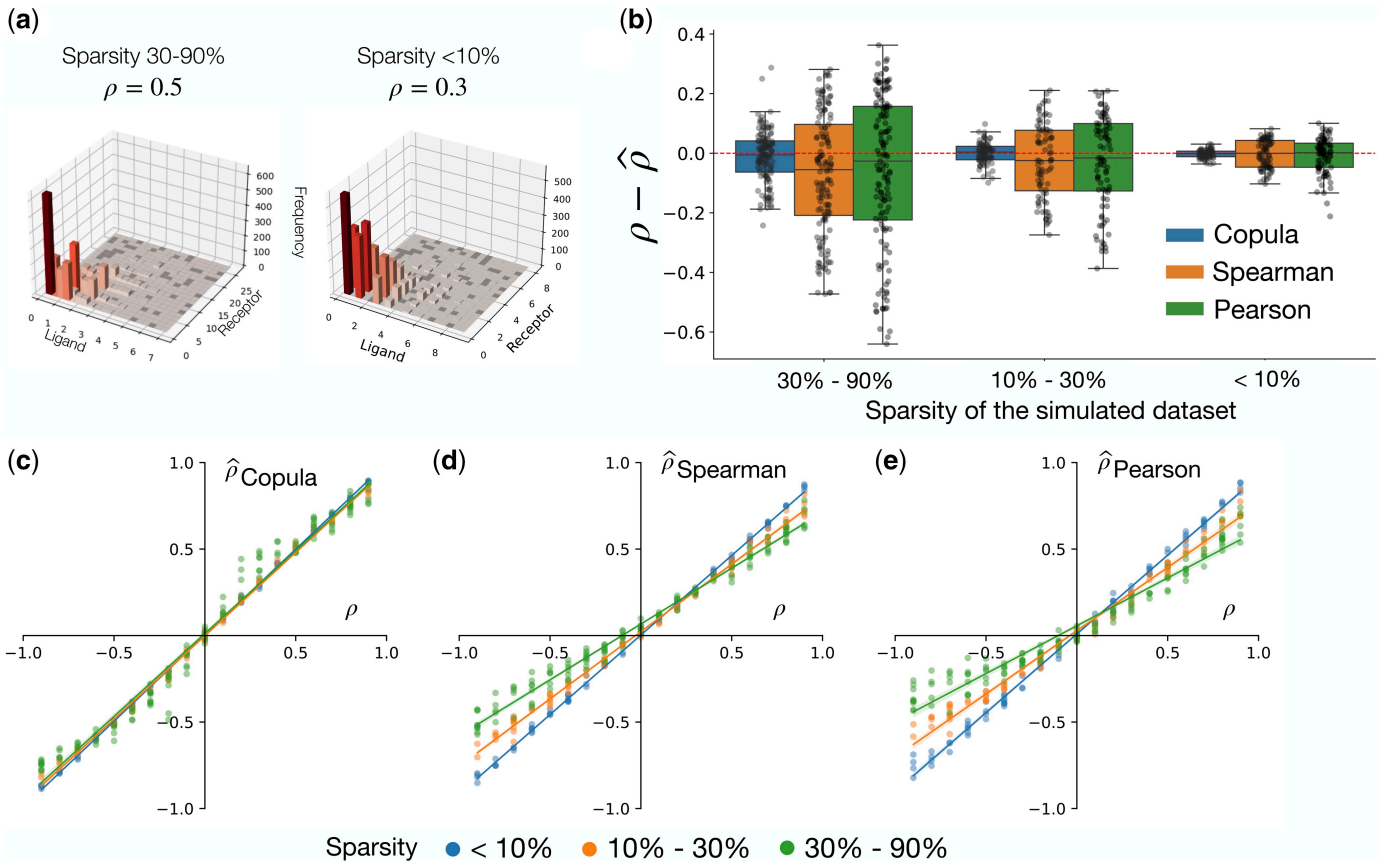
Evaluation of Copulacci on simulated data. **(a)** 3D histogram of simulated ligand–receptor expression counts $\left(\ell_{i},r_{j}\right)$ with correlation ρ and different levels of data sparsity. The *x*-axis is the ligand expression $\ell_{i}$; the *y*-axis is the receptor expression $r_{j}$; and the height of each bar is the observed number of pairs $\left(\ell_{i},r_{j}\right)$ in the simulated data. **(b)** Difference $\hat{\rho }-\rho$ between true correlation ρ and predicted correlation $\hat{\rho }$ for Copulacci (Copula), Spearman correlation coefficient, and Pearson correlation coefficient, across different simulated instances, grouped by sparsity of simulated ligand and receptor expression values. **(c–e)** True correlation ρ versus estimated **(c)** copula correlation coefficient (${\hat{\rho }}_{\text{Copula}}$) learned by Copulacci, **(d)** Spearman correlation coefficient (${\hat{\rho }}_{\text{Spearman}}$), and **(e)** Pearson correlation coefficient (${\hat{\rho }}_{\text{Pearson}}$) across different simulated instances. Lines are colored according to data sparsity and are fit by a linear regression.

We first evaluated Copulacci using simulated non-spatial count data. We drew 500 samples $\left(\ell_{i},r_{j}\right)$ of ligand and receptor expression values from a bivariate Gaussian copula distribution with Poisson marginals, where the bivariate Gaussian has a covariance matrix $\Sigma_{p}=\left(\begin{array}{cc} 1 & \rho \\ \rho & 1 \end{array}\right)$ and the Poisson marginals are $\ell_{i}∼\text{Pois}(kN_{i}e^{\mu_{\ell }})$ and $r_{j}∼\text{Pois}(kN_{j}e^{\mu_{r}})$ for the ligand and receptor, respectively. The parameters $N_{i}$ represent the total UMI count at a spot *i*, and we set it equal to the total UMI counts from a 10x Visium dataset (Xu *et al.* 2022, Bergenstråhle *et al.* 2020). The parameter *k* controls the sparsity of the simulated data, such that the ligand and receptor expressions $\left(\ell_{i},r_{j}\right)$ are more sparse for smaller values of *k* and vice versa (Fig. 2a). We choose the sparsity parameter *k* such that the data sparsity falls into three buckets: <10%, 10%–30%, and more than 30%. See Supplementary Appendix for more details.

We compare the copula correlation coefficient estimated by Copulacci to the Pearson and Spearman correlation coefficients, which are standard correlation measures used to quantify CCIs in single-cell and spatial analysis (Armingol *et al.* 2021). In particular, we computed the Spearman and Pearson correlation coefficients on log-transformed and normalized UMI counts, as is standard in the literature (Li *et al.* 2023). We note that we do not compare to the Moran’s I measure as we do not simulate spatial locations.

Copulacci more accurately estimates the true correlation coefficient ρ compared to the Spearman and Pearson correlation coefficients for all parameter choices (Fig. 2b). In particular, Copulacci has the most improvement over the Pearson and Spearman correlations when the ligand and receptor UMI counts have sparsity of at least 30%, i.e. a similar level of sparsity as many current SRT technologies. We also emphasize that the Copulacci accuracy has noticeably smaller variance compared to the accuracy of the Spearman and Pearson correlation coefficients. This demonstrates that Copulacci is more robust than the Pearson and Spearman correlation measure, especially on sparse data.

Moreover, we observe that the Pearson and Spearman correlations tend to *underestimate* the true absolute correlation coefficient |ρ| (Fig. 2c–e). In particular, when there is a large amount of data sparsity (>30%) and the magnitude of the correlation coefficient ρ is large (i.e. |ρ|>0.5), then the Spearman and Pearson correlations have a much smaller magnitude than the true correlation coefficient, i.e. $\left|\hat{\rho }|\;\ll |\rho \right|$. In contrast, Copulacci estimates a correlation coefficient ${\hat{\rho }}_{\text{Copula}}$ with a magnitude close to the true correlation coefficient ρ across all data sparsity levels and coefficients ρ.

These simulations demonstrate that, by using a count-based model, Copulacci more accurately measures correlations between ligands and receptors in sparse SRT data.

### 3.2 Visium data from human breast cancer

We next used Copulacci to infer CCIs from SRT data of a human breast cancer (invasive ductal carcinoma) measured with 10x Genomics Visium (Xu *et al.* 2022, Bergenstråhle *et al.* 2020). In this dataset, we observe the expression of 649 ligands and receptors—forming 1315 pairs of interacting ligands and receptors, as obtained from (Li *et al.* 2023)—in 3798 spots. The data exhibits a high level of sparsity, with the expression of a given ligand or receptor occurring in merely 25% of these locations on average.

Since there is no ground truth, we compare the correlation coefficients ${\hat{\rho }}_{\text{Copula}}$ estimated by Copulacci to those estimated by two other methods, MERINGUE (Miller *et al.* 2021) and SpatialDM (Li *et al.* 2023). MERINGUE and SpatialDM estimate variants of the generalized Pearson correlation, which MERINGUE calls spatial cross-correlation (see Section 2). Moreover, since each spot in this dataset is annotated with one of four cell type labels (Xu *et al.* 2022)—tumor, surrounding tumor, invasive region, and healthy—we analyze cell type-specific CCIs (see Supplementary Table S1), i.e. CCIs between adjacent spots with two pre-specified cell types.

**Figure 3.**
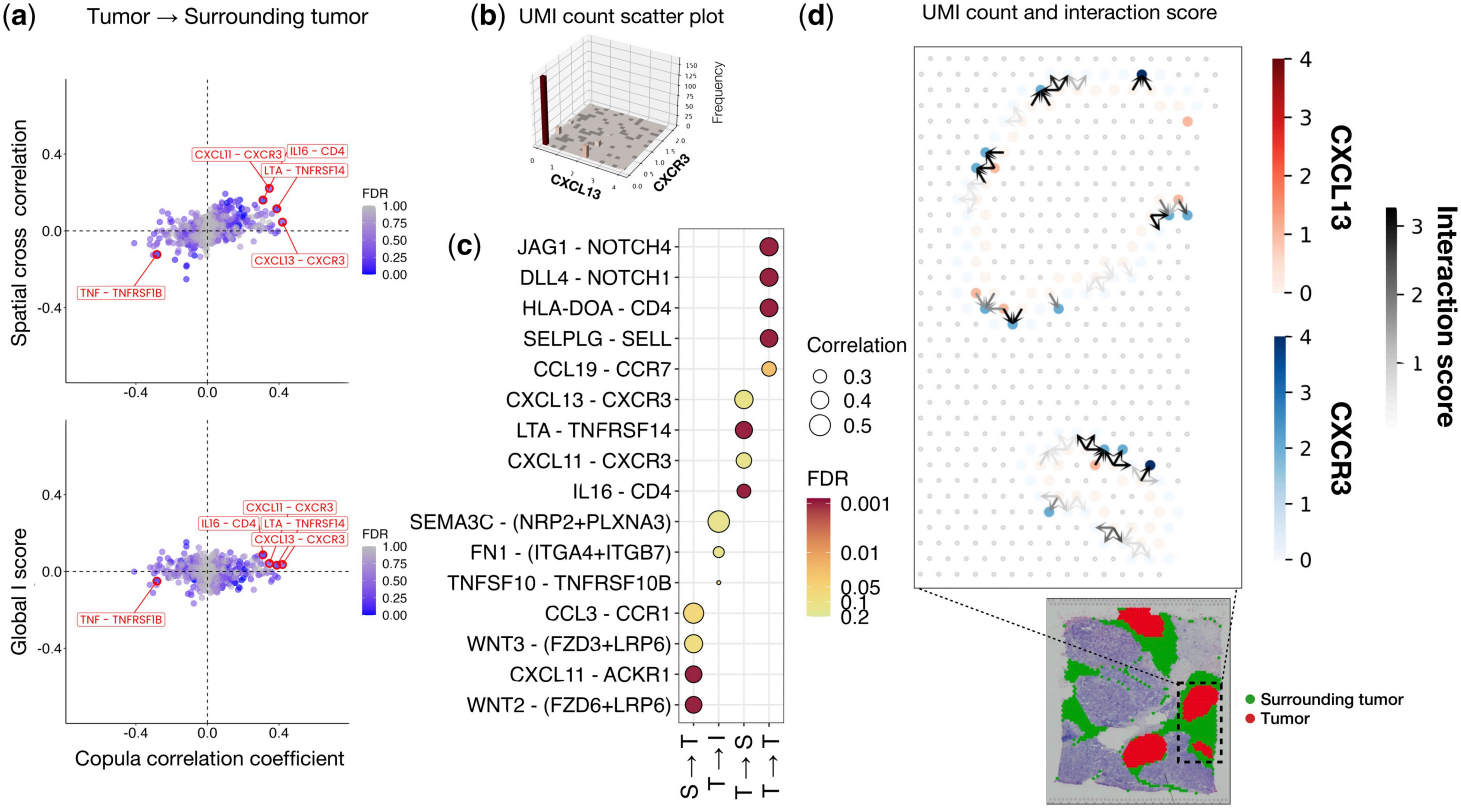
Ligand–receptor interactions inferred from Copulacci in a breast cancer SRT dataset. **(a)** Scatter plot of the copula correlation coefficient (from Copulacci) versus: (top) the spatial cross-correlation coefficient used by MERINGUE (Miller *et al.* 2021) and (bottom) the global I score used by SpatialDM (Li *et al.* 2023) for ligand–receptor pairs (ℓ,r) from tumor to surrounding tumor cell types. The five ligand–receptor pairs highlighted have the largest absolute difference between correlation coefficients. **(b)** 3D histogram plot of ligand UMI count versus receptor UMI count for ligand–receptor pair *CXCL13*-*CXCR3*. **(c)** Dotplot highlighting the statistically significant ligand–receptor pairs across cell type interactions where one of the participating cell type is tumor. Cell types are abbreviated as tumor (T), surrounding tumor (S), and invasive (I). Color indicates FDR corrected *p*-values from the Copulacci permutation test. **(d)** UMI counts and Copulacci directed interaction score for the ligand–receptor pair *CXCL13*-*CXCR3* for spots at the border of the tumor and surrounding tumor regions in the boxed region of the tissue slice.

We observe that Copulacci estimates markedly different correlation coefficients compared to SpatialDM and MERINGUE for CCIs between tumor and surrounding tumor cell types (Fig. 3a). In particular, Copulacci estimates substantially larger correlation coefficients than both SpatialDM and MERINGUE. We also find that the ligand–receptor pairs in which Copulacci estimates a larger correlation than SpatialDM or MERINGUE tend to have sparser expression, for example the ligand–receptor pairs *IL16*-*CD4* and *CXCL13*-*CXCR3* (Fig. 3b). Since SpatialDM and MERINGUE use variants of the Pearson correlation coefficients, these results align with our simulation results—where we showed that Pearson correlation underestimates the true correlation for sparse data.

The ligand–receptor pairs with large correlations estimated by Copulacci are involved in several important tumor processes. For example, Copulacci identifies the ligand–receptor pairs *CXCL13*-*CXCR3* and *CXCL11*-*CXCR3* as having a significantly (false discovery rate <0.2) large correlation (${\hat{\rho }}_{\text{Copula}}=0.41$ and ${\hat{\rho }}_{\text{Copula}}=0.39$, respectively) between tumor and surrounding tumor spots (Fig. 3c). The ligand proteins *CXCL13* and *CXCL11* and receptor protein *CXCR3* are chemokines whose interactions are known to mediate the immune response in the tumor microenvironment, e.g. interactions between *CXCL13* and *CXCR3* recruit suppressive immune cells into tumors in some cancer types (Gao *et al.* 2021, Gao and Zhang 2021). Another ligand–receptor pair that Copulacci identifies as having a significantly large correlation is *IL16*-*CD4*. *IL16* is a chemoattractant, and increased *IL16* expression has been observed to recruit *CD4*+ pro-tumor macrophages in different breast cancers (Richmond *et al.* 2014, Hridi *et al.* 2021, Mathy *et al.* 2000). We note that existing tools, including SpatialDM and MERINGUE, do not identify these ligand–receptor pairs while Copulacci does.

Copulacci uses the Mahalanobis distance to quantify the interaction strength between a ligand and a receptor at different spatial locations (see Section 2), enabling the visualization of where CCIs take place in a tissue. For example, Copulacci determines the regions at the interface between the tumor and surrounding tumor regions where the interactions between *CXCL13* and *CXCR3* occur (Fig. 3d). This localization may lead to the identification of biologically interesting spatial cohorts or niches that can be dissected more closely. We emphasize that MERINGUE is unable to visualize these interactions, while SpatialDM is only able to identify large neighborhoods where interactions take place, not individual spatial locations.

Overall, our analysis shows that Copulacci measures accurate and biologically meaningful CCIs in the tumor microenvironment.

### 3.3 SeqFISH+ data from mouse cortex

We next applied Copulacci to SRT of the mouse somatosensory cortex measured with seqFISH+ (Eng *et al.* 2019). In this dataset, the expression of 1157 ligand–receptor pairs are measured in 523 spots. Here, each measured spot is a single cell, in contrast to 10x Visium where each spot contains multiple cells. This SeqFISH+ dataset is less sparse than 10x Visium data: on average, a ligand or receptor is expressed in approximately 30% of the spots.

Copulacci identified several biologically meaningful CCIs between the L5 eNeurons, L6 eNeurons, and astrocytes in the mouse cortex (Supplementary Fig. S1a). In particular, Copulacci identifies several CCIs between astrocytes and L6 eNeurons mediated by ligands and receptors in the *Wnt* signaling pathway, which is known to regulate many neuronal processes in the mouse cortex including neuronal migration (Bocchi *et al.* 2017) and synaptic plasticity (Folke *et al.* 2019). We also highlight the interaction (Supplementary Fig. S1b) between the laminin ligand *Lamc3* and the heteromeric integrin receptor *Itgav* + *Itgb8*, which has a substantially larger copula correlation coefficient compared to the correlation coefficients computed by MERINGUE and SpatialDM (Supplementary Fig. S2). Mutations to integrin proteins have been observed to result in abnormal laminar organization of the mouse cortex, suggesting that interactions between laminins and integrins are crucial for mouse cortex function (Anton *et al.* 1999). This analysis demonstrates how Copulacci reveals biologically meaningful CCIs in highly sparse SRT data which are missed by current approaches.

### 3.4 Stereo-seq data from a mouse embryo

We used Copulacci to infer CCIs from SRT of a mouse embryo (embryonic day E9.5) measured with Stereo-seq (Chen *et al.* 2022). Copulacci identifies several biologically meaningful interacting ligand–receptor pairs in the mouse embryo that are missed by existing methods (Supplementary Fig. S3), including ligand–receptor pairs at the interface between the brain and neural crest. See Supplementary Appendix for details.

## 4 Discussion

The rapid development of SRT technologies opens new avenues for the systematic analysis of high-resolution molecular mechanisms such as CCIs. However, the analysis of current spatial SRT data is challenged by low UMI counts. Existing approaches use correlation measures designed for continuous-valued data and are unable to accurately measure correlations between discrete and sparse UMI counts. Copulacci resolves this problem by directly modeling the raw UMI counts from ligands and receptors using a Gaussian copula model, which can model dependencies in discrete data and accounts for sparsity using Poisson marginals. We showed that Copulacci outperforms the standard correlation measures using both simulated data and real SRT data. In particular, we show that standard correlation measures based on Pearson or Moran’s I tend to underestimate the true correlation. As a result, Copulacci identifies many novel CCIs that are missed by existing approaches. Moreover, Copulacci’s copula model also allows for the identification of spatial regions where a CCI is taking place.

There are several future directions for Copulacci. First, a major limitation of every existing CCI inference methods—including Copulacci—is the lack of a ground truth in most real biological tissues. Thus, it would be useful to apply Copulacci to different samples of the same tissue type, e.g. using different technologies, and create “consensus” lists of probable CCIs that can be used to benchmark CCI inference methods. Second, Copulacci and other CCI inference methods rely on a database of candidate ligand–receptor interactions (Efremova *et al.* 2020, Jin *et al.* 2021), which potentially biases algorithms toward ligands and receptors that have been previously studied. It would be useful to extend Copulacci to identify interacting ligands and receptors *de novo* from sparse SRT data. Third, one could extend Copulacci to model dependencies between other counts derived from SRT data. For example, for technologies with low spatial resolution such as 10x Genomics Visium, it may be useful to model correlations between counts of *cell types* (e.g. derived from Kleshchevnikov *et al.* 2022) using a copula with binomial marginals. Finally, the count-based model in Copulacci could be extended to model differential interactions across various cell states and cell types in multiple case–control samples, further elucidating the CCIs responsible for shaping the spatial organization of tissues.

## Supplementary Material

btae219_Supplementary_Data

## Data Availability

Code and data were available at https://github.com/raphael-group/copulacci.

## References

[btae219-B1] 10x Genomics. *10x Visium Genomics Visium Spatial Gene Expression*. https://www.10xgenomics.com/products/spatial-gene-expression (25 January 2024, date last accessed).

[btae219-B2] Amprino R. Cellular interactions in cell differentiation. Development 1953;1:283–5.

[btae219-B3] Anton E , Kreidberg JA, Rakic P. Distinct functions of α3 and αv integrin receptors in neuronal migration and laminar organization of the cerebral cortex. Neuron 1999;22:277–89. 10069334 10.1016/s0896-6273(00)81089-2

[btae219-B4] Armingol E , Officer A, Harismendy O et al Deciphering cell–cell interactions and communication from gene expression. Nat Rev Genet 2021;22:71–88. 33168968 10.1038/s41576-020-00292-x PMC7649713

[btae219-B5] Arnol D , Schapiro D, Bodenmiller B et al Modeling cell-cell interactions from spatial molecular data with spatial variance component analysis. Cell Rep 2019;29:202–11.e6. 31577949 10.1016/j.celrep.2019.08.077 PMC6899515

[btae219-B6] Baccin C , Al-Sabah J, Velten L et al Combined single-cell and spatial transcriptomics reveal the molecular, cellular and spatial bone marrow niche organization. Nat Cell Biol 2020;22:38–48. 31871321 10.1038/s41556-019-0439-6 PMC7610809

[btae219-B7] Bafna M , Li H, Zhang X. Clarify: cell–cell interaction and gene regulatory network refinement from spatially resolved transcriptomics. Bioinformatics 2023;39:i484–i493. 37387180 10.1093/bioinformatics/btad269 PMC10311313

[btae219-B8] Baker SG. The multinomial-poisson transformation. Journal of the Royal Statistical Society: Series D (the Statistician) 1994;43:495–504.

[btae219-B9] Benjamini Y , Hochberg Y. Controlling the false discovery rate: a practical and powerful approach to multiple testing. Journal of the Royal Statistical Society: series B (Methodological) 1995;57:289–300.

[btae219-B10] Bergenstråhle J , Larsson L, Lundeberg J. Seamless integration of image and molecular analysis for spatial transcriptomics workflows. BMC Genomics 2020;21:482–7. 32664861 10.1186/s12864-020-06832-3 PMC7386244

[btae219-B11] Bishara AJ , Hittner JB. Testing the significance of a correlation with nonnormal data: comparison of pearson, spearman, transformation, and resampling approaches. Psychol Methods 2012;17:399–417. 22563845 10.1037/a0028087

[btae219-B12] Bocchi R , Egervari K, Carol-Perdiguer L et al Perturbed wnt signaling leads to neuronal migration delay, altered interhemispheric connections and impaired social behavior. Nat Commun 2017;8:1158. 29079819 10.1038/s41467-017-01046-w PMC5660087

[btae219-B13] Booeshaghi AS , Pachter L. Normalization of single-cell rna-seq counts by log (x+ 1) or log (1+ x). Bioinformatics 2021;37:2223–4. 33676365 10.1093/bioinformatics/btab085 PMC7989636

[btae219-B14] Cabello-Aguilar S , Alame M, Kon-Sun-Tack F et al Singlecellsignalr: inference of intercellular networks from single-cell transcriptomics. Nucleic Acids Res 2020;48:e55. 32196115 10.1093/nar/gkaa183 PMC7261168

[btae219-B15] Campbell J. The poisson correlation function. Proceedings of the Edinburgh Mathematical Society 1934;4:18–26.

[btae219-B16] Cang Z , Nie Q. Inferring spatial and signaling relationships between cells from single cell transcriptomic data. Nat Commun 2020;11:2084. 32350282 10.1038/s41467-020-15968-5 PMC7190659

[btae219-B17] Cang Z , Zhao Y, Almet AA et al Screening cell–cell communication in spatial transcriptomics via collective optimal transport. Nat Methods 2023;20:218–28. 36690742 10.1038/s41592-022-01728-4 PMC9911355

[btae219-B18] Chen A , Liao S, Cheng M et al Spatiotemporal transcriptomic atlas of mouse organogenesis using dna nanoball-patterned arrays. Cell 2022;185:1777–92.e21. 35512705 10.1016/j.cell.2022.04.003

[btae219-B19] Chitra U , Arnold BJ, Sarkar H et al Mapping the topography of spatial gene expression with interpretable deep learning. bioRxiv, 2023. 10.1101/2023.10.10.561757. PMC12370209 39849132

[btae219-B20] Choi H , Sheng J, Gao D et al Transcriptome analysis of individual stromal cell populations identifies stroma-tumor crosstalk in mouse lung cancer model. Cell Rep 2015;10:1187–201. 25704820 10.1016/j.celrep.2015.01.040

[btae219-B21] Dries R , Zhu Q, Dong R et al Giotto: a toolbox for integrative analysis and visualization of spatial expression data. Genome Biol 2021;22:78–31. 33685491 10.1186/s13059-021-02286-2 PMC7938609

[btae219-B22] Efremova M , Vento-Tormo M, Teichmann SA et al Cellphonedb: inferring cell–cell communication from combined expression of multi-subunit ligand–receptor complexes. Nat Protoc 2020;15:1484–506. 32103204 10.1038/s41596-020-0292-x

[btae219-B23] Eng C-HL , Lawson M, Zhu Q et al Transcriptome-scale super-resolved imaging in tissues by RNA seqfish+. Nature 2019;568:235–9. 30911168 10.1038/s41586-019-1049-y PMC6544023

[btae219-B24] Folke J , Pakkenberg B, Brudek T. Impaired wnt signaling in the prefrontal cortex of Alzheimer’s disease. Mol Neurobiol 2019;56:873–91. 29804228 10.1007/s12035-018-1103-z

[btae219-B25] Gao Q , Zhang Y. Cxcl11 signaling in the tumor microenvironment. Adv Exp Med Biol. 2021;1302:41–50 34286440 10.1007/978-3-030-62658-7_4

[btae219-B26] Gao S-H , Liu S-Z, Wang G-Z et al Cxcl13 in cancer and other diseases: biological functions, clinical significance, and therapeutic opportunities. Life 2021;11:1282. 34947813 10.3390/life11121282 PMC8708574

[btae219-B27] Genest C , Nešlehová J. A primer on copulas for count data. ASTIN Bull 2007;37:475–515.

[btae219-B28] Getis A , Ord JK. The analysis of spatial association by use of distance statistics. Geographical Analysis 1992;24:189–206.

[btae219-B29] Hafemeister C , Satija R. Normalization and variance stabilization of single-cell rna-seq data using regularized negative binomial regression. Genome Biol 2019;20:296. 31870423 10.1186/s13059-019-1874-1 PMC6927181

[btae219-B30] Hou R , Denisenko E, Ong HT et al Predicting cell-to-cell communication networks using natmi. Nat Commun 2020;11:5011. 33024107 10.1038/s41467-020-18873-z PMC7538930

[btae219-B31] Hridi SU , Barbour M, Wilson C et al Increased levels of il-16 in the Central nervous system during neuroinflammation are associated with infiltrating immune cells and resident glial cells. Biology (Basel) 2021;10:472. 34071825 10.3390/biology10060472 PMC8229350

[btae219-B32] Hu Y , Peng T, Gao L et al Cytotalk: de novo construction of signal transduction networks using single-cell transcriptomic data. Sci Adv 2021;7:eabf1356. 33853780 10.1126/sciadv.abf1356 PMC8046375

[btae219-B33] Inouye DI , Yang E, Allen GI et al A review of multivariate distributions for count data derived from the poisson distribution. WIREs Comput Stat 2017;9:e1398. 10.1002/wics.1398 PMC5624559 28983398

[btae219-B34] Jin S , Guerrero-Juarez CF, Zhang L et al Inference and analysis of cell-cell communication using cellchat. Nat Commun 2021;12:1088. 33597522 10.1038/s41467-021-21246-9 PMC7889871

[btae219-B35] Kazianka H , Pilz J. Copula-based geostatistical modeling of continuous and discrete data including covariates. Stoch Environ Res Risk Assess 2010;24:661–73.

[btae219-B36] Kirouac DC , Ito C, Csaszar E et al Dynamic interaction networks in a hierarchically organized tissue. Mol Syst Biol 2010;6:417. 20924352 10.1038/msb.2010.71 PMC2990637

[btae219-B37] Kleshchevnikov V , Shmatko A, Dann E et al Cell2location maps fine-grained cell types in spatial transcriptomics. Nat Biotechnol 2022;40:661–71. 35027729 10.1038/s41587-021-01139-4

[btae219-B38] Kveler K , Starosvetsky E, Ziv-Kenet A et al Immune-centric network of cytokines and cells in disease context identified by computational mining of pubmed. Nat Biotechnol 2018;36:651–9. 29912209 10.1038/nbt.4152 PMC6035104

[btae219-B39] Leineweber W. Integrated biophysical imaging of cell interactions with 3d extracellular matrices. Nat Rev Mol Cell Biol 2023;24:773–1. 10.1038/s41580-023-00639-2 37402840

[btae219-B40] Li Z , Wang T, Liu P et al Spatialdm for rapid identification of spatially co-expressed ligand–receptor and revealing cell–cell communication patterns. Nat Commun 2023;14:3995. 37414760 10.1038/s41467-023-39608-w PMC10325966

[btae219-B41] Lu P , Weaver VM, Werb Z. The extracellular matrix: a dynamic niche in cancer progression. J Cell Biol 2012;196:395–406. 22351925 10.1083/jcb.201102147 PMC3283993

[btae219-B42] Ma C , Chitra U, Zhang S et al Belayer: modeling discrete and continuous spatial variation in gene expression from spatially resolved transcriptomics. Cell Syst 2022;13:786–97.e13. 36265465 10.1016/j.cels.2022.09.002 PMC9814896

[btae219-B43] Madsen L. Maximum likelihood estimation of regression parameters with spatially dependent discrete data. JABES 2009;14:375–91.

[btae219-B44] Mathy N , Scheuer W, Lanzendörfer M et al Interleukin-16 stimulates the expression and production of pro-inflammatory cytokines by human monocytes. Immunology 2000;100:63–9. 10809960 10.1046/j.1365-2567.2000.00997.x PMC2326980

[btae219-B45] Miller BF , Bambah-Mukku D, Dulac C et al Characterizing spatial gene expression heterogeneity in spatially resolved single-cell transcriptomic data with nonuniform cellular densities. Genome Res 2021;31:1843–55. 34035045 10.1101/gr.271288.120 PMC8494224

[btae219-B46] Miller EJ , Lappin SL. *Physiology, Cellular Receptor*. In: StatPearls [Internet]. Treasure Island (FL): StatPearls Publishing 2020. 32119290

[btae219-B47] Mitchell C , Paulson A. A new bivariate negative binomial distribution. Naval Research Logistics 1981;28:359–74.

[btae219-B48] Nishida-Aoki N , Gujral TS. Emerging approaches to study cell–cell interactions in tumor microenvironment. Oncotarget 2019;10:785–97. 30774780 10.18632/oncotarget.26585 PMC6366828

[btae219-B49] O'Hara R , Kotze J. Do not log-transform count data. Nat Prec 2010;118–122.

[btae219-B50] Palla G , Spitzer H, Klein M et al Squidpy: a scalable framework for spatial omics analysis. Nat Methods 2022;19:171–8. 35102346 10.1038/s41592-021-01358-2 PMC8828470

[btae219-B51] Pham D , Tan X, Xu J et al Stlearn: integrating spatial location, tissue morphology and gene expression to find cell types, cell-cell interactions and spatial trajectories within undissociated tissues. Nat Commun 2023;14:7739.

[btae219-B52] Rao A , Barkley D, França GS et al Exploring tissue architecture using spatial transcriptomics. Nature 2021;596:211–20. 34381231 10.1038/s41586-021-03634-9 PMC8475179

[btae219-B53] Richmond J , Tuzova M, Cruikshank W et al Regulation of cellular processes by interleukin-16 in homeostasis and cancer. J Cell Physiol 2014;229:139–47. 23893766 10.1002/jcp.24441

[btae219-B54] Rivera LB , Bergers G. Tumor angiogenesis, from foe to friend. Science 2015;349:694–5. 26273044 10.1126/science.aad0862

[btae219-B55] Rüschendorf L , Rüschendorf L. Copulas, sklar’s theorem, and distributional transform. In: Mathematical Risk Analysis: Springer Series in Operations Research and Financial Engineering. Berlin, Heidelberg: Springer, 2013, 3–34.

[btae219-B56] Skinner MK. Cell-cell interactions in the testis. Endocr Rev 1991;12:45–77. 2026122 10.1210/edrv-12-1-45

[btae219-B57] Song D , Wang Q, Yan G et al scdesign3 generates realistic in silico data for multimodal single-cell and spatial omics. Nat Biotechnol 2023;42:247–52. 37169966 10.1038/s41587-023-01772-1 PMC11182337

[btae219-B58] Subrahmaniam K , Subrahmaniam K. On the estimation of the parameters in the bivariate negative binomial distribution. Journal of the Royal Statistical Society: Series B (Methodological) 1973;35:131–46.

[btae219-B59] Sun T , Song D, Li WV et al scdesign2: a transparent simulator that generates high-fidelity single-cell gene expression count data with gene correlations captured. Genome Biol 2021;22:163. 34034771 10.1186/s13059-021-02367-2 PMC8147071

[btae219-B60] Townes FW , Hicks SC, Aryee MJ et al Feature selection and dimension reduction for single-cell rna-seq based on a multinomial model. Genome Biol 2019;20:295. 31870412 10.1186/s13059-019-1861-6 PMC6927135

[btae219-B61] Türei D , Valdeolivas A, Gul L et al Integrated intra-and intercellular signaling knowledge for multicellular omics analysis. Mol Syst Biol 2021;17:e9923. 33749993 10.15252/msb.20209923 PMC7983032

[btae219-B62] Wang Y , Wang R, Zhang S et al Italk: an r package to characterize and illustrate intercellular communication. bioRxiv, 10.1101/507871, 2019, preprint.

[btae219-B63] Williams CG , Lee HJ, Asatsuma T et al An introduction to spatial transcriptomics for biomedical research. Genome Med 2022;14:68–18. 35761361 10.1186/s13073-022-01075-1 PMC9238181

[btae219-B64] Wu H , Malone AF, Donnelly EL et al Single-cell transcriptomics of a human kidney allograft biopsy specimen defines a diverse inflammatory response. J Am Soc Nephrol 2018;29:2069–80. 29980650 10.1681/ASN.2018020125 PMC6065085

[btae219-B65] Xu C , Jin X, Wei S et al Deepst: identifying spatial domains in spatial transcriptomics by deep learning. Nucleic Acids Res 2022;50:e131. 36250636 10.1093/nar/gkac901 PMC9825193

