# Supplementary material for: A count-based model for delineating cell–cell interactions in spatial transcriptomics data
Source: Bioinformatics. 2024 Jun 28;40(Suppl 1):i481–9. doi: 10.1093/bioinformatics/btae219 (PMC11211854; doi:10.1093/bioinformatics/btae219)
Supplement: btae219_Supplementary_Data [file btae219_supplementary_data.zip › btae219_Supplementary_Data/Raphael.265.sup.5.pdf]

Appendix

Stereo-seq data from a mouse embryo

We next used Copulacci to infer CCIs from spatial transcriptomics of a mouse embryo (embryonic day E9.5) measured with Stereo-seq (Chen et al., 2022). This data measures the expression of 593 ligands and receptors, which form $1,401$ pairs of interacting ligands and receptors, in $5,913$ spots. In contrast to 10x Visium, where spots contain multiple cells, in Stereo-seq each measured spot is a single cell. However, Stereo-seq data is noticeably sparser than 10x Visium data. In this dataset, only 12% of spots express the given ligands or receptors.

We applied Copulacci to this dataset to identify CCIs responsible for organogenesis in the mouse embryo. Specifically, we used Copulacci to learn cell type-specific CCIs that involve the neural crest cell type, which is a cell type that gives rise to many different types of cells including muscle cells, bone cells, and neurons (Huang and Saint-Jeannet, 2004) and whose interactions with other cell types are critical for cellular differentiation (Takahashi et al., 2013).

Copulacci identifies several biologically meaningful interacting ligand-receptor pairs in the mouse embryo (Figure S3a). For example, the most significant ligand receptor pairs at the interface between the brain and neural crest for which Copulacci estimates a larger correlation $\widehat{\rho}_{\text{Copula}}$ compared to MERINGUE and SpatialDM (Figure S4) include *Lama1-Sv2a*, *Lama1-Sv2a*, *Lama4-Cd44*. All these interactions belong to the laminin signaling pathway, an essential pathway responsible for neural crest cell migration and formation during embryogenesis (Miner et al., 1998). The interaction score computed by Copulacci shows that the interactions between *Lamb1* and *Sv2a* occur throughout the interface (Figure S3b) between the brain and neural crest cell types. We highlight that the extreme sparsity of the Stereo-seq dataset poses a significant challenge for current approaches to identify biologically relevant CCIs. Copulacci, however, successfully uncovers these interactions, demonstrating its capability in analyzing sparse datasets.

Simulation set-up

Our count simulation (Sun et al., 2021) involves three steps. Given a correlation coefficient ρ, the Poisson mean parameters μ_ℓ, μ_r, UMI counts N_i, and sparsity parameter k, the process is as follows:

- Step 1: Given a target correlation coefficient ρ, sample a set of variables $(z_{i,\ell}, z_{j,r}) \sim \mathcal{N}(0, \Sigma_\rho)$ from a bivariate standard normal distribution with covariance matrix $\Sigma_\rho = \begin{pmatrix} 1 & \rho \\ \rho & 1 \end{pmatrix}$.
- Step 2: Compute $(\Phi_{\mathcal{N}(0,1)}(z_{i,\ell}), \Phi_{\mathcal{N}(0,1)}(z_{j,r}))$, where $\Phi_{\mathcal{N}(0,1)}$ is the CDF of a standard normal distribution $\mathcal{N}(0,1)$.
- Step 3: Apply the inverse CDF for the Poisson distribution with individual ligand and receptor mean μ_ℓ and μ_r under the GLM-poisson framework. Then the count values are obtained using $\ell_i = F_L^{-1}(\Phi(z_{i,\ell}))$ and $r_j = F_R^{-1}(\Phi(z_{j,r}))$, where $F_L(\ell)$, and $F_R(r)$, are the CDFs of the Poisson distributions $\text{Pois}(kN_i e^{\mu_\ell})$ and $\text{Pois}(lN_j e^{\mu_r})$, respectively.

The raw UMI counts N_i are taken from a real Visium experiment (Xu et al., 2022; Bergenstråhle et al., 2020) with 3460 spots. We induce desired sparsity in the simulated datasets with the parameter k. In summary we vary the parameters k, μ_ℓ, μ_r, ρ with $\rho \in [-0.9, 0.9]$, $\mu_\ell, \mu_r \in [-9, -3]$ and $k \in \{0.005, 0.01, 0.05, 0.8, 0.1\}$ to sample 500 ligand-receptor pairs for 3460 spots. We further stratified these simulated ligand receptor expression pairs into three groups based on the percentage of zeroes present in the their respective expression vector.

We vary the parameters k, μ_ℓ, μ_r, ρ with $\rho \in [-0.9, 0.9]$, $\mu_\ell, \mu_r \in [-9, -3]$ and $k \in \{0.005, 0.01, 0.05, 0.8, 0.1\}$ to sample 500 ligand-receptor pairs.

Distributional transform

The distributional transform involves transforms a discrete CDF F to a continuous one by using the transformation $F'(a_i) = uF(a_i - 1) + (1 - u)F(a_i)$ where u is sampled form a uniform distribution $U(0,1)$. This transformation requires multiple rounds of sampling to estimate the CDF and could be computationally challenging, therefore we use the simplified version as proposed in (Kazianka and Pilz, 2010). Namely, we use $F'(a_i) = 0.5(F(a_i - 1) + F(a_i))$ which takes the average of the CDF for the two end points of a discrete interval.

Dataset description

We used Copulacci to infer CCIs from three different datasets that are generated using different spatial transcriptomics technologies: (1) human breast cancer (Invasive Ductal Carcinoma) spatial transcriptomics data from the 10X Genomics Visium platform, termed as IDC Visium data (Xu et al., 2022; Bergenstråhle et al., 2020); (2) mouse somatosensory cortex measured with seqFISH+ (Eng et al., 2019) (3) sagittal section from C57BL/6 mouse embryonic day E9.5 spatial transcriptomics data, obtained using the Stereo-seq technology, termed as Stereo-seq data (Chen et al., 2022). Visium, seqFISH+, and Stereo-seq have different spatial resolution, number of expressed genes, and depth of expression. For example, the IDC Visium dataset contains a large gene panel, yet the expression of genes is often sparse, moreover the resolution of an individual spot is approximately 50μm, indicating one spot may contain multiple cells. On the other hand Stereo-seq dataset provides sub-cellular resolution of 220nm enabling close approximation of single-cell spatial spots after computational preprocessing. SeqFISH+ also provides spots with single-cell resolution with each spot measuring 10,000 genes.

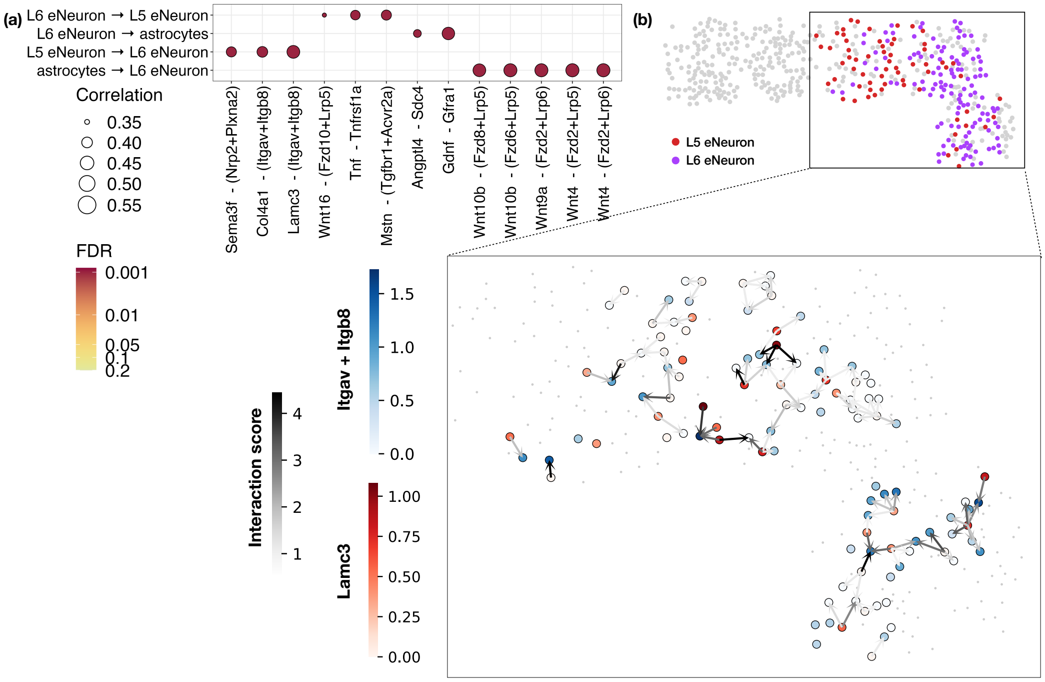

Fig. S1. Ligand-receptor interactions inferred from Copulacci in a seqFISH+ mouse cortex dataset. (a) Dotplot highlighting the statistically significant ligand-receptor pairs identified by Copulacci, for interactions between the L5 eNeuron cell type to L6 eNeuron cell type. The five ligand-receptor pairs with the highest estimated correlation coefficient $\hat{\rho}_{\text{Copula}}$ are shown. The color indicate the FDR-corrected P-values from the Copulacci permutation test. **(b)** Location of L5 eNeuron (red) and L6 eNeuron (violet) cell types in the mouse cortex. Boxed region highlights the boundary between L5 eNeuron and L6 eNeuron cell types. The log-normalized expression of individual genes ligand-receptor pair Lamc3 - (Itgav + Itgb8), along with the corresponding interaction score computed by Copulacci.

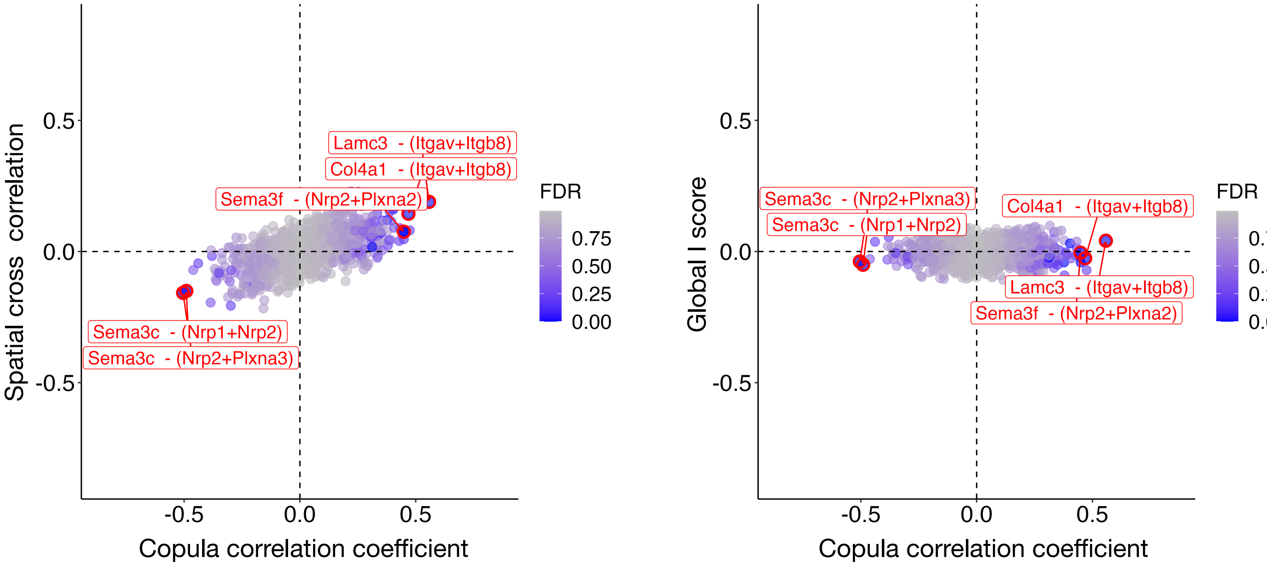

Fig. S2. The scatter compares Copula correlation coefficient (from Copulacci) against (left) the spatial cross correlation coefficient used by MERINGUE (Miller et al., 2021) and (right) the global I score used by SpatialDM (Li et al., 2023) focusing on the ligand-receptor pairs from L5 eNeuron to L6 eNeuron in seqFISH dataset . The top 5 significant (FDR ≤ 0.1) ligand-receptor pairs are labeled based on the largest absolute differences between the correlation coefficient estimated by Copulacci and those obtained by MERINGUE and SpatialDM

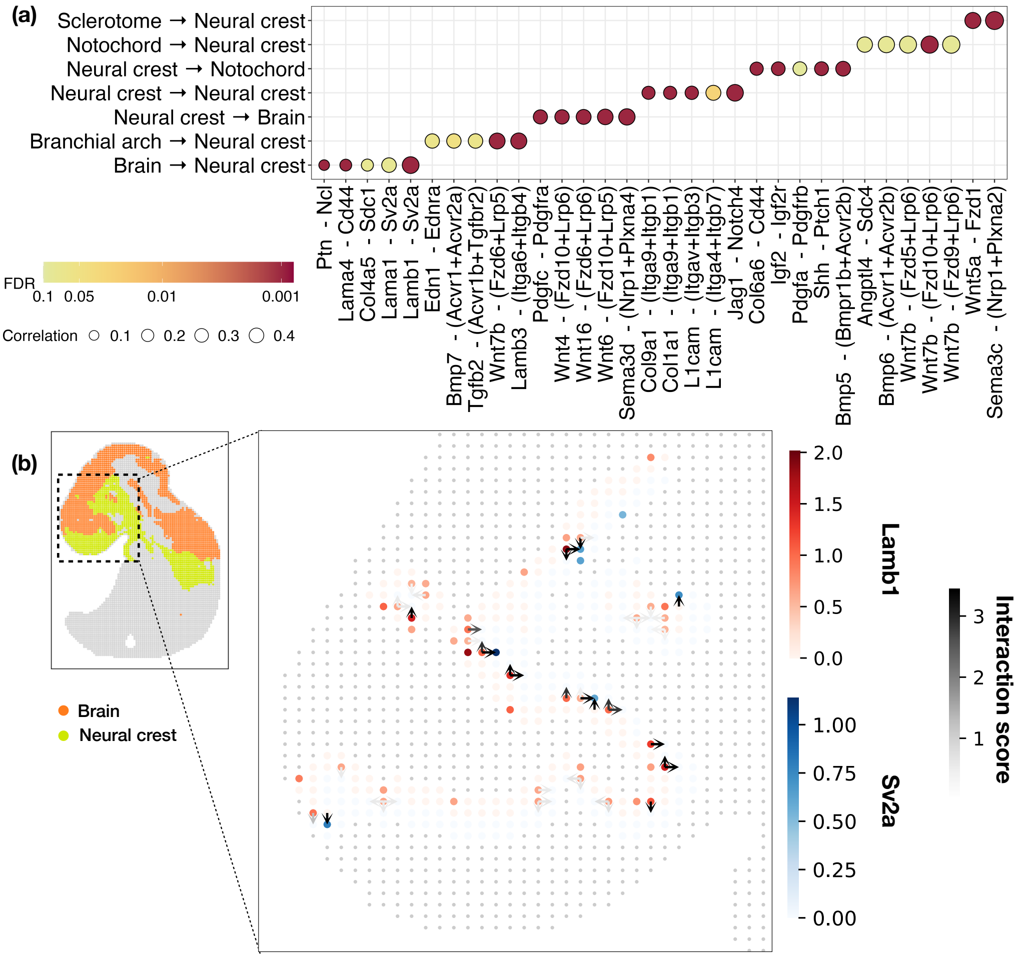

Fig. S3. Ligand-receptor interactions inferred from Copulacci in a mouse organogenesis Stereo-seq dataset. (a) Dotplot highlighting the statistically significant ligand-receptor pairs identified by Copulacci, for interactions between the neural crest cell type and all other cell types. The five ligand-receptor pairs with the highest estimated correlation coefficient $\hat{\rho}_{\text{Copula}}$ are shown. The color indicate the FDR-corrected P-values from the Copulacci permutation test. **(b)** Location of brain (orange) and neural crest (yellow) cell types in the mouse embryo. Boxed region highlights the boundary between the brain and the neural crest cell type. The log-normalized expression of individual genes ligand-receptor pair Lamb1-Sv2a, along with the corresponding interaction score computed by Copulacci.

Source	Target	Spots	Edges	Average ligand UMI count	Average receptor UMI count
Surrounding tumor	Surrounding tumor	823	1768	1.39	1.41
Invasive	Invasive	2027	6418	2.01	2.95
Healthy	Healthy	485	1438	0.45	0.47
Tumor	Tumor	463	1307	1.54	2.69
Surrounding tumor	Invasive	610	615	1.53	2.28
Invasive	Surrounding tumor	610	615	1.69	1.73
Tumor	Invasive	34	27	1.79	2.52
Invasive	Tumor	34	27	1.94	2.24
Tumor	Surrounding tumor	244	234	2.00	2.23
Surrounding tumor	Tumor	244	234	2.33	3.06
Healthy	Surrounding tumor	170	154	0.60	0.82
Surrounding tumor	Healthy	170	154	0.78	0.67
Healthy	Invasive	60	55	0.71	1.69
Invasive	Healthy	60	55	1.14	0.73
Tumor	Healthy	26	23	2.15	1.23
Healthy	Tumor	26	23	1.18	2.72

Table S1. Summary statistics related to cell type specific CCIs categorized by ligand cell type and receptor cell type. For each ligand cell type and receptor cell type each row contains the number of spots participating, number of edges, average ligand UMI counts and average receptor UMI counts.

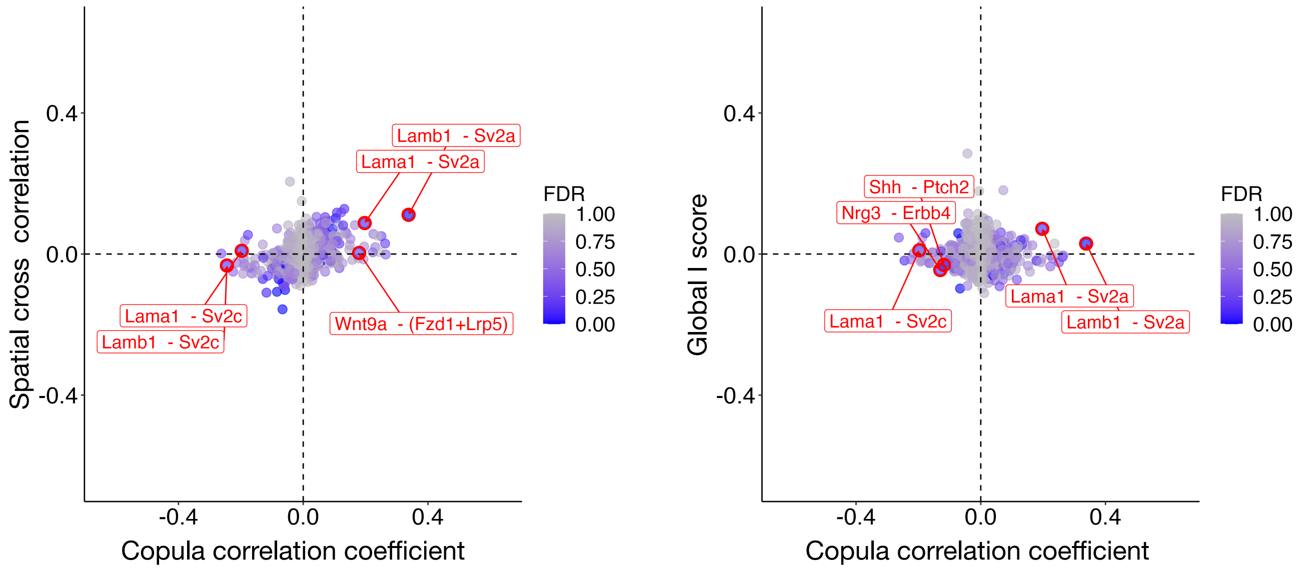

Fig. S4. The scatter compares Copula correlation coefficient (from Copulacci) against (left) the spatial cross correlation coefficient used by MERINGUE (Miller et al., 2021) and (right) the global I score used by SpatialDM (Li et al., 2023) focusing on the ligand-receptor pairs from brain to neural crest CCIs. The top 5 significant (FDR ≤ 0.2) ligand-receptor pairs are labeled based on the and largest absolute differences between the correlation coefficient estimated by Copulacci and those obtained by MERINGUE and SpatialDM

References

J. Bergenstråhle, L. Larsson, and J. Lundeberg. Seamless integration of image and molecular analysis for spatial transcriptomics workflows. *BMC genomics*, 21(1):1–7, 2020.

A. Chen, S. Liao, M. Cheng, K. Ma, L. Wu, Y. Lai, X. Qiu, J. Yang, J. Xu, S. Hao, et al. Spatiotemporal transcriptomic atlas of mouse organogenesis using dna nanoball-patterned arrays. *Cell*, 185(10):1777–1792, 2022.

C.-H. L. Eng, M. Lawson, Q. Zhu, R. Dries, N. Koulena, Y. Takei, J. Yun, C. Cronin, C. Karp, G.-C. Yuan, et al. Transcriptome-scale super-resolved imaging in tissues by rna seqfish+. *Nature*, 568(7751):235–239, 2019.

X. Huang and J.-P. Saint-Jeannet. Induction of the neural crest and the opportunities of life on the edge. *Developmental biology*, 275(1):1–11, 2004.

H. Kazianka and J. Pilz. Copula-based geostatistical modeling of continuous and discrete data including covariates. *Stochastic environmental research and risk assessment*, 24:661–673, 2010.

Z. Li, T. Wang, P. Liu, and Y. Huang. Spatialdm for rapid identification of spatially co-expressed ligand–receptor and revealing cell–cell communication patterns. *Nature Communications*, 14(1):3995, 2023.

B. F. Miller, D. Bambah-Mukku, C. Dulac, X. Zhuang, and J. Fan. Characterizing spatial gene expression heterogeneity in spatially resolved single-cell transcriptomic data with nonuniform cellular densities. *Genome research*, 31(10):1843–1855, 2021.

J. H. Miner, J. Cunningham, and J. R. Sanes. Roles for laminin in embryogenesis: exencephaly, syndactyly, and placentopathy in mice lacking the laminin $\alpha 5$ chain. *The Journal of cell biology*, 143(6):1713–1723, 1998.

T. Sun, D. Song, W. V. Li, and J. J. Li. scdesign2: a transparent simulator that generates high-fidelity single-cell gene expression count data with gene correlations captured. *Genome biology*, 22(1):163, 2021.

Y. Takahashi, D. Sipp, and H. Enomoto. Tissue interactions in neural crest cell development and disease. *Science*, 341(6148): 860–863, 2013.

C. Xu et al. Deepst: identifying spatial domains in spatial transcriptomics by deep learning. *Nucleic Acids Research*, 50(22): e131–e131, 2022.
