# Supplementary figures and images for: A count-based model for delineating cell–cell interactions in spatial transcriptomics data
Source: Bioinformatics. 2024 Jun 28;40(Suppl 1):i481–9. doi: 10.1093/bioinformatics/btae219 (PMC11211854; doi:10.1093/bioinformatics/btae219)

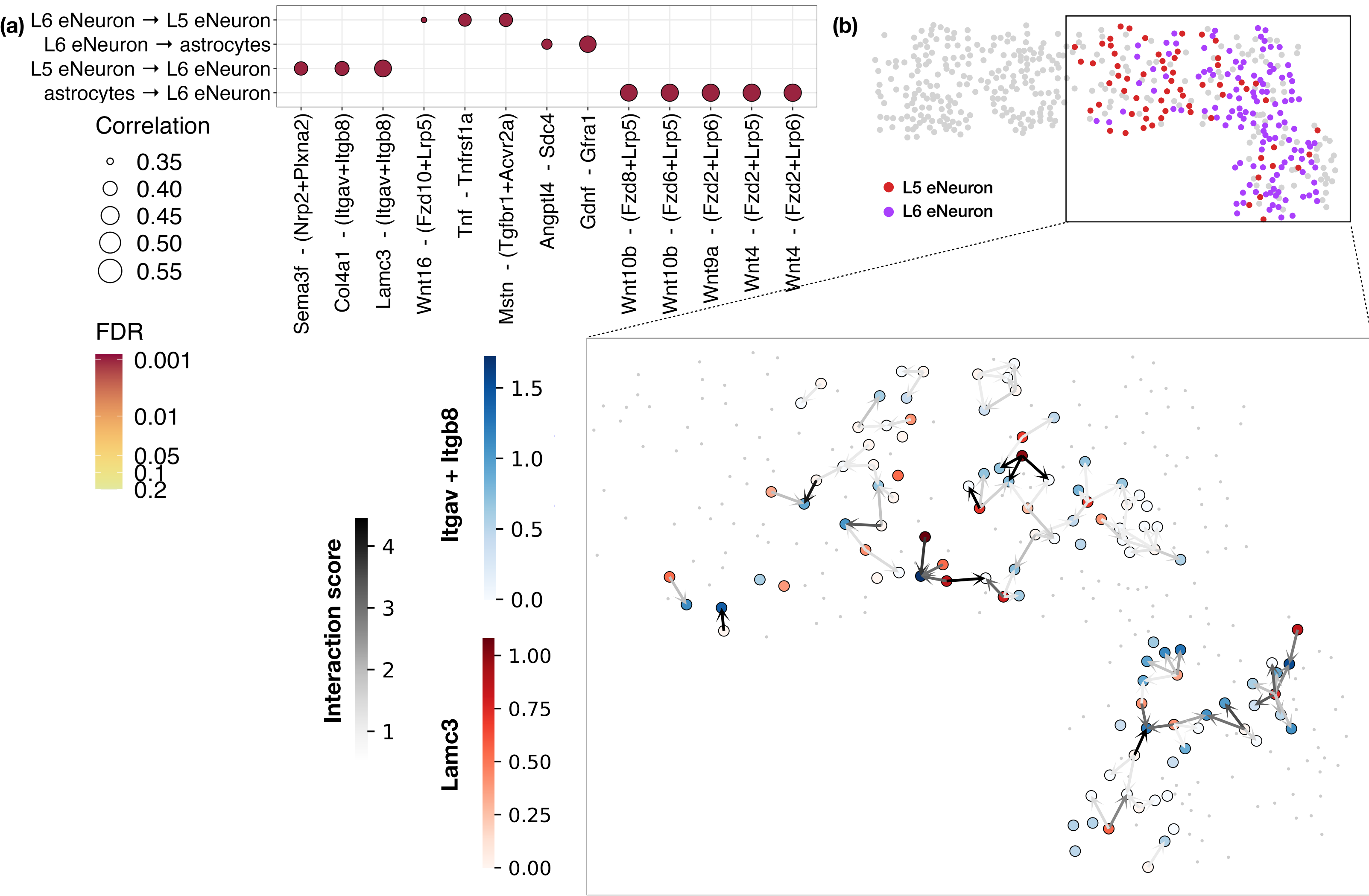

Supplement: btae219_Supplementary_Data [file btae219_supplementary_data.zip › btae219_Supplementary_Data/Raphael.265.sup.1.pdf]

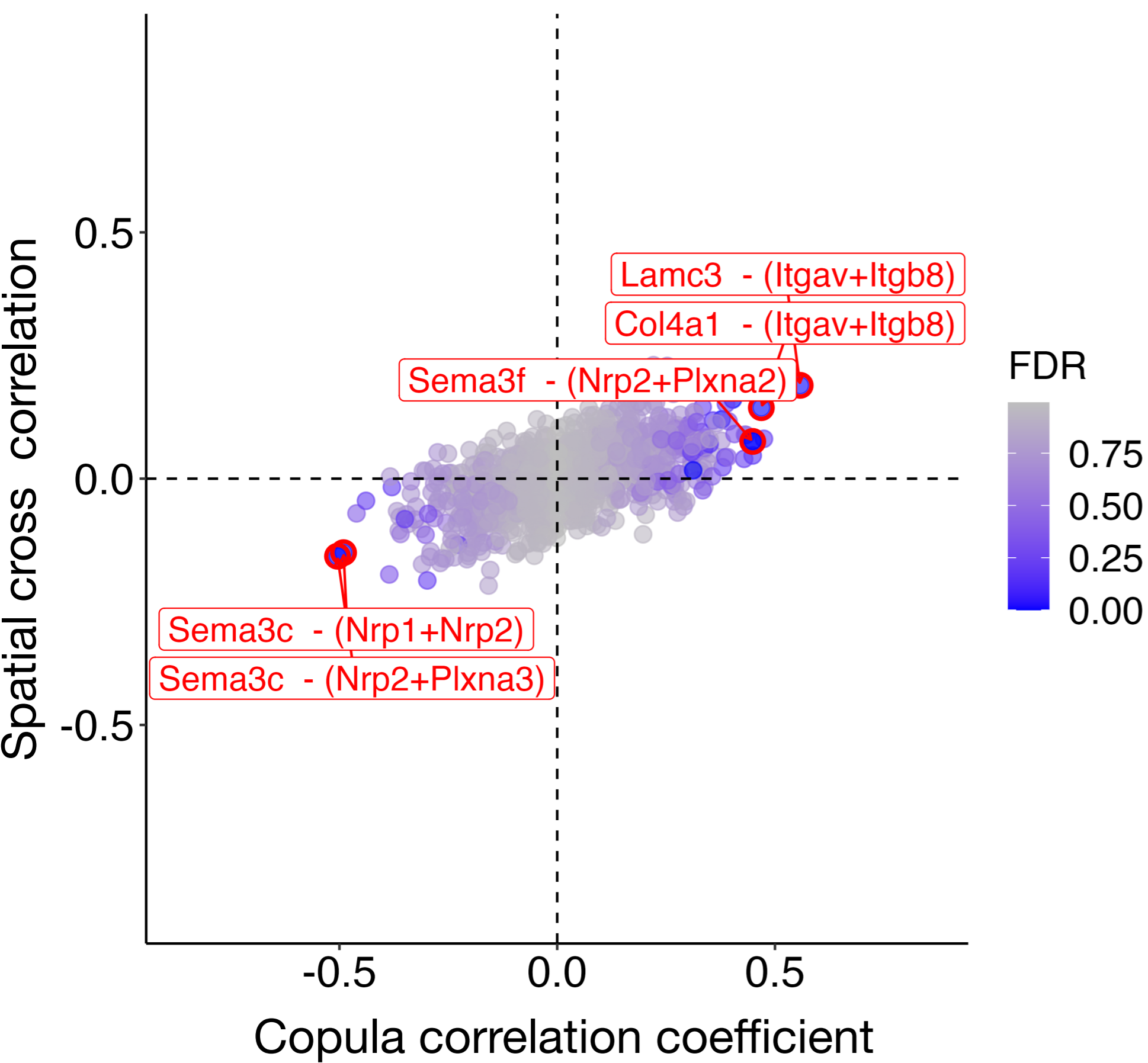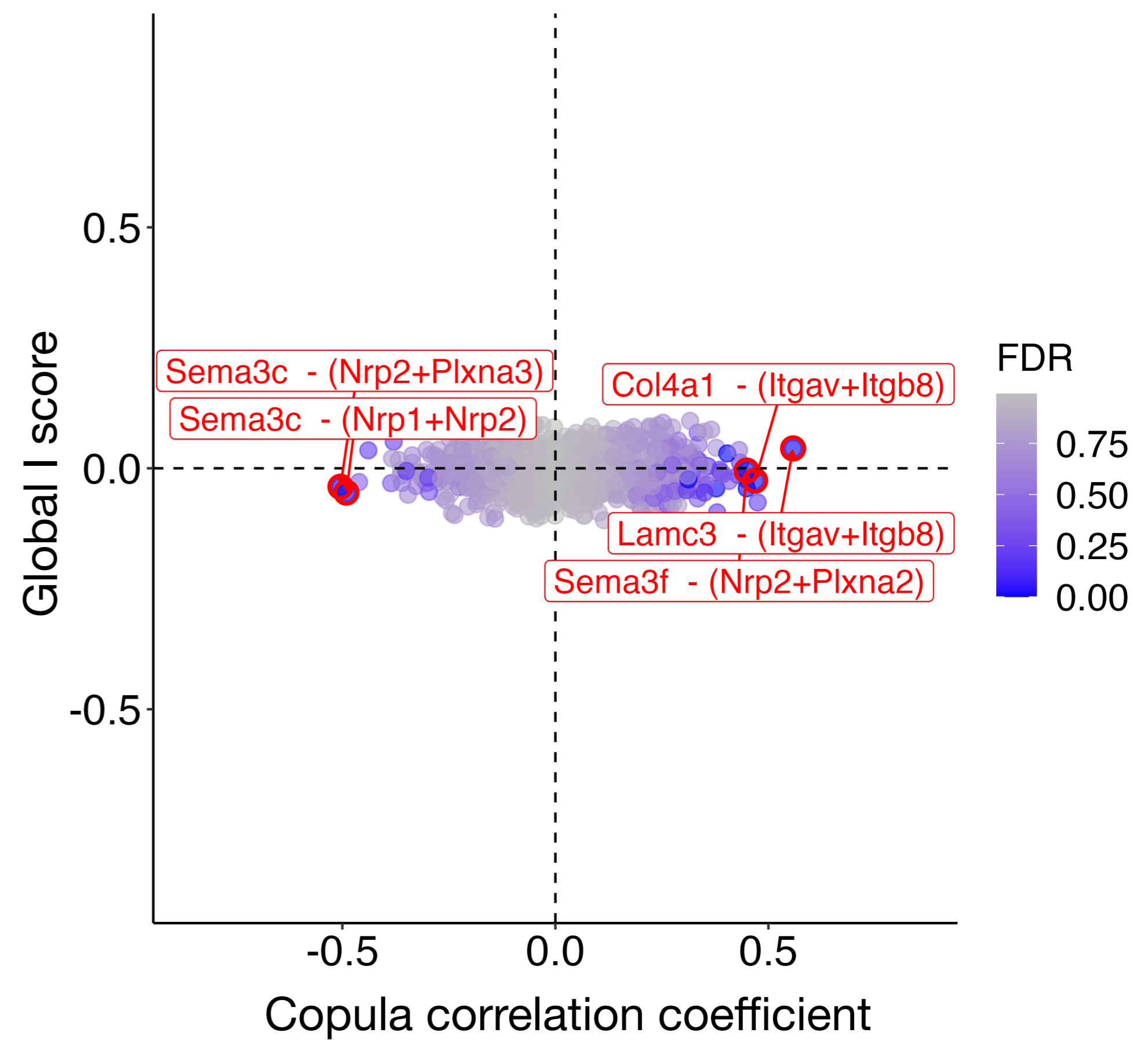

Supplement: btae219_Supplementary_Data [file btae219_supplementary_data.zip › btae219_Supplementary_Data/Raphael.265.sup.2.pdf]

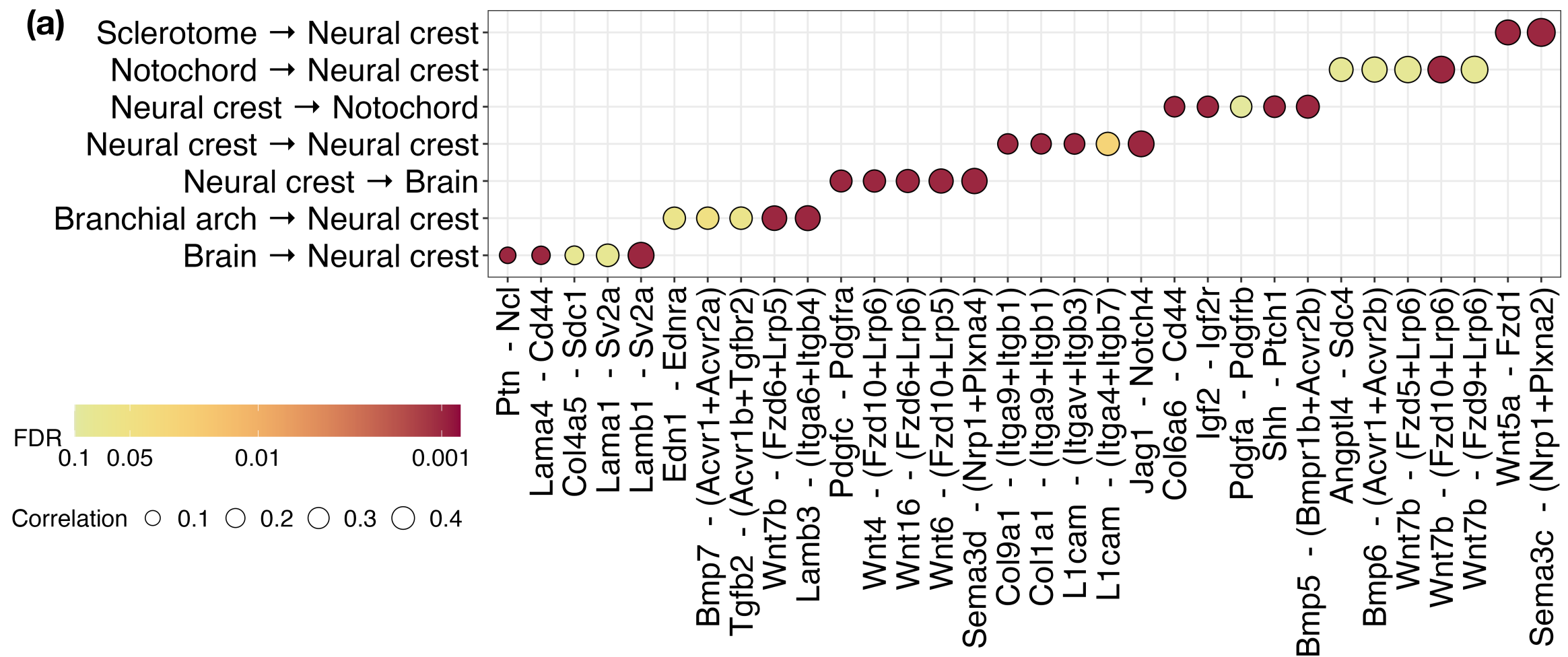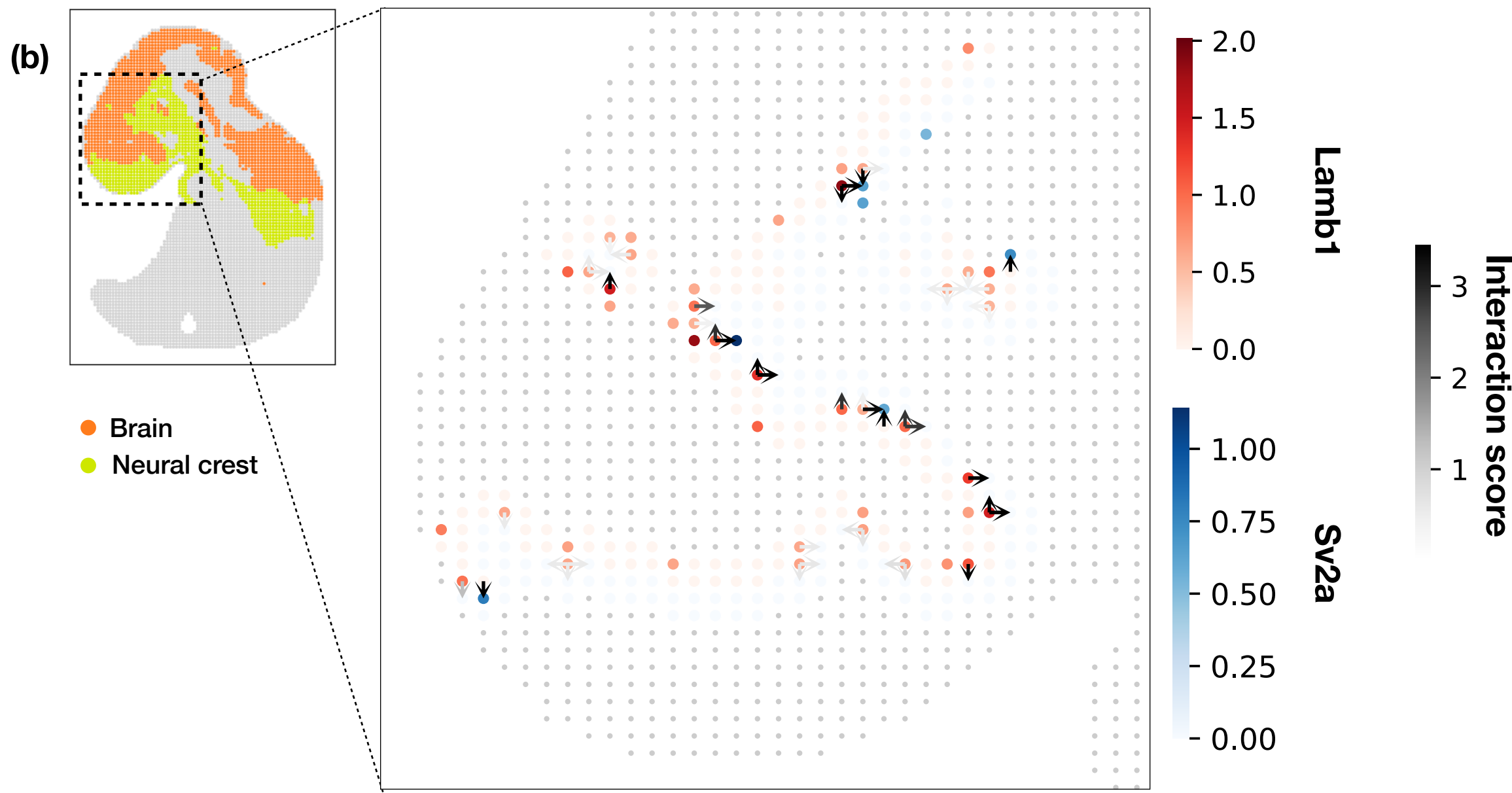

Supplement: btae219_Supplementary_Data [file btae219_supplementary_data.zip › btae219_Supplementary_Data/Raphael.265.sup.3.pdf]

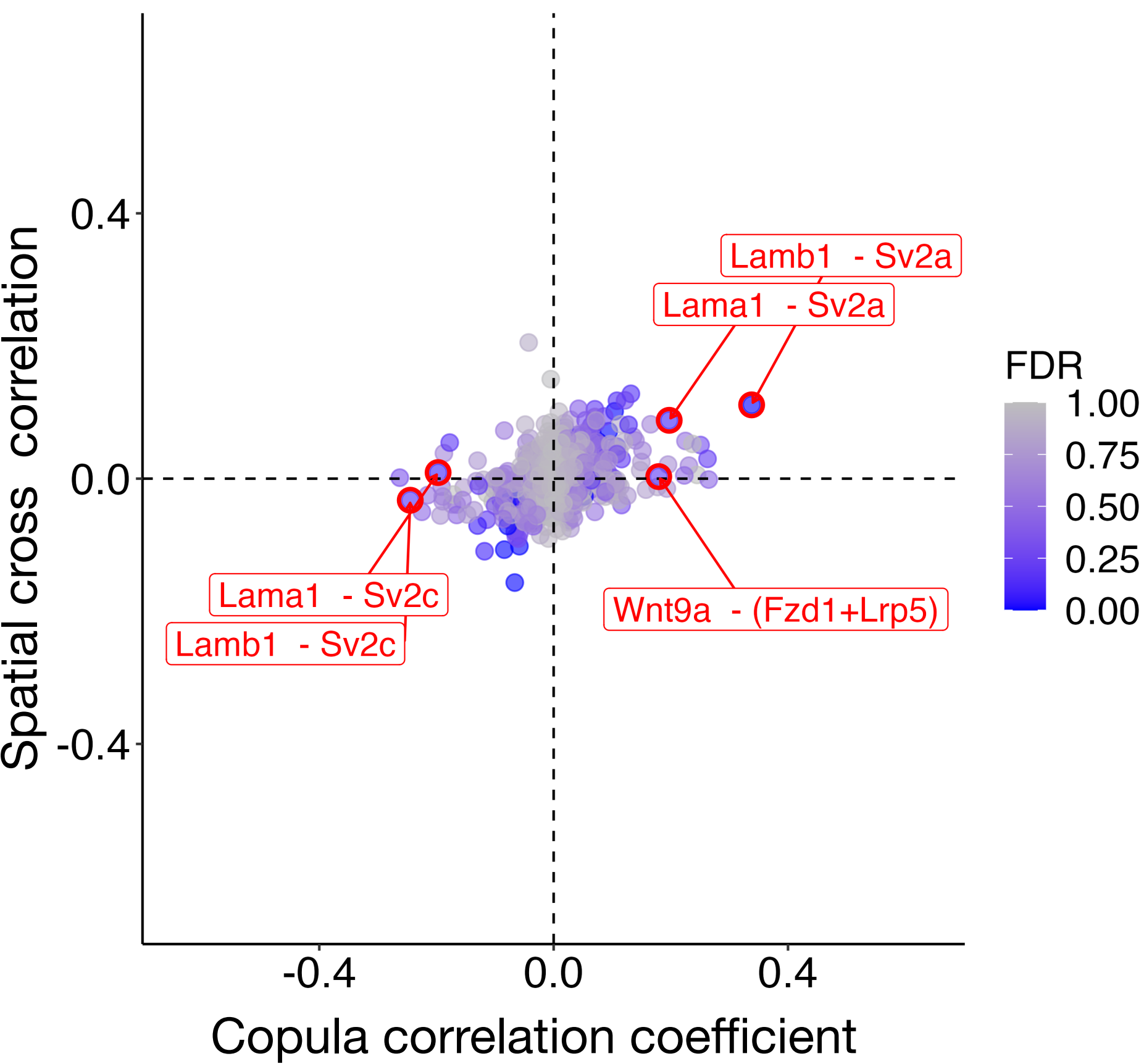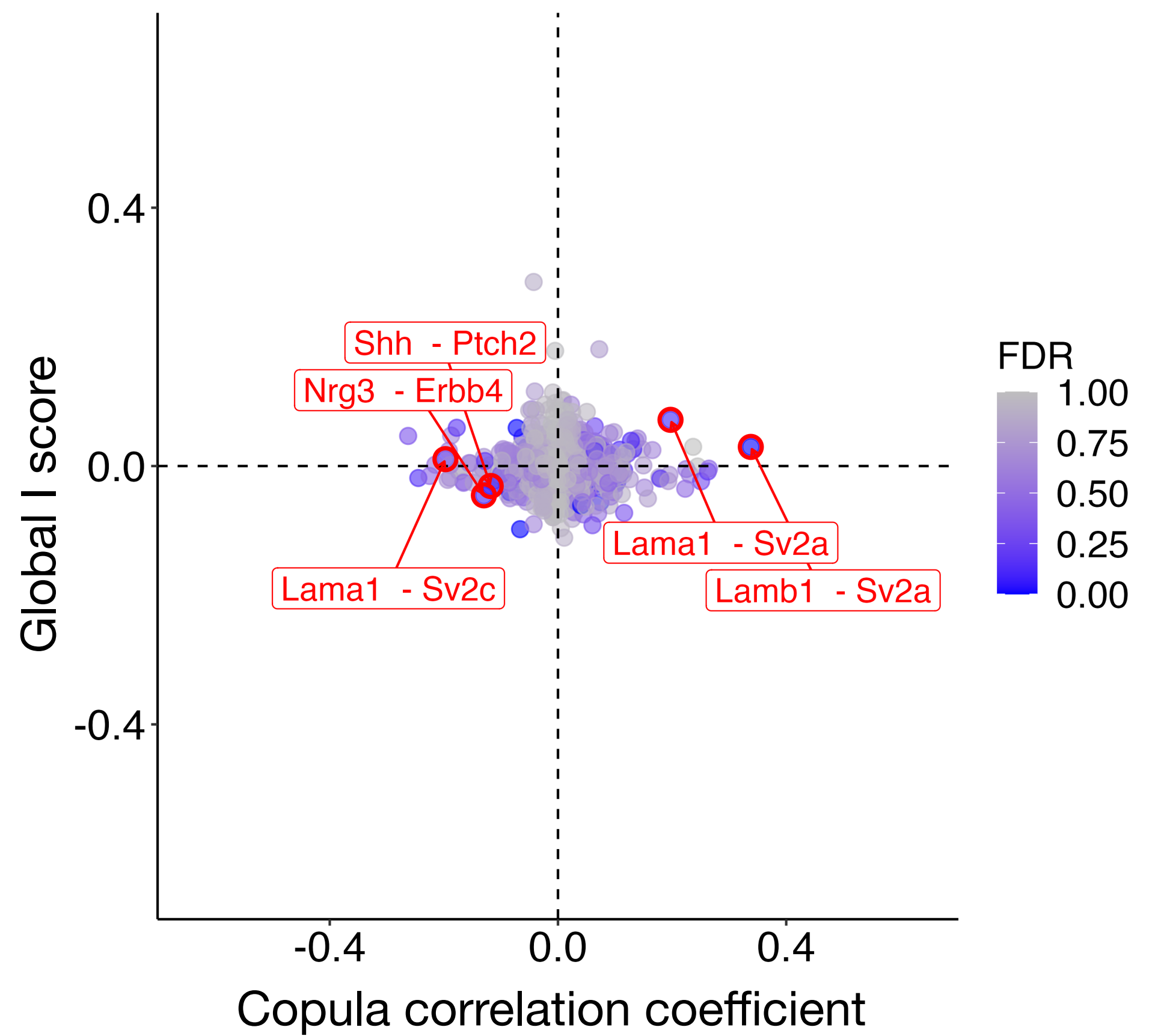

Supplement: btae219_Supplementary_Data [file btae219_supplementary_data.zip › btae219_Supplementary_Data/Raphael.265.sup.4.pdf]
